# Zika virus infection as a cause of congenital brain abnormalities and Guillain-Barré syndrome: From systematic review to living systematic review

**DOI:** 10.12688/f1000research.13704.1

**Published:** 2018-02-15

**Authors:** Michel Jacques Counotte, Dianne Egli-Gany, Maurane Riesen, Million Abraha, Teegwendé Valérie Porgo, Jingying Wang, Nicola Low

**Affiliations:** 1 Institute of Social and Preventive Medicine, University of Bern, Bern, Switzerland; 2 Department of Social and Preventative Medicine, Université Laval, Québec, Canada

**Keywords:** Zika virus, causality, living systematic review, congenital abnormalities, Guillain-barre syndrome, microcephlay

## Abstract

**Background. **The Zika virus (ZIKV) outbreak in the Americas has caused international concern due to neurological sequelae linked to the infection, such as microcephaly and Guillain-Barré syndrome (GBS). The World Health Organization stated that there is “sufficient evidence to conclude that Zika virus is a cause of congenital abnormalities and is a trigger of GBS”. This conclusion was based on a systematic review of the evidence published until 30.05.2016. Since then, the body of evidence has grown substantially, leading to this update of that systematic review with new evidence published from 30.05.2016 – 18.01.2017, update 1.

**Methods. **We review evidence on the causal link between ZIKV infection and adverse congenital outcomes and the causal link between ZIKV infection and GBS or immune-mediated thrombocytopaenia purpura. We also describe the transition of the review into a living systematic review, a review that is continually updated.

**Results. **Between 30.05.2016 and 18.01.2017, we identified 2413 publications, of which 101 publications were included. The evidence added in this update confirms the conclusion of a causal association between ZIKV and adverse congenital outcomes. New findings expand the evidence base in the dimensions of biological plausibility, strength of association, animal experiments and specificity. For GBS, the body of evidence has grown during the search period for update 1, but only for dimensions that were already populated in the previous version. There is still a limited understanding of the biological pathways that potentially cause the occurrence of autoimmune disease following ZIKV infection.

**Conclusions. **This systematic review confirms previous conclusions that ZIKV is a cause of congenital abnormalities, including microcephaly, and is a trigger of GBS. The transition to living systematic review techniques and methodology provides a proof of concept for the use of these methods to synthesise evidence about an emerging pathogen such as ZIKV.

## Introduction

Outbreaks of Zika virus (ZIKV) infection in the Americas have caused international concern owing to the severity of neurological sequelae linked to the infection ( WHO statement IHR 2005). During 2016, the number of countries affected by the ZIKV outbreak had grown from 33 countries ( WHO situation report 05.02.2016) to 75 countries ( WHO situation report 05.01.2017). By March 9, 2017, 31 countries had reported microcephaly or other congenital central nervous system (CNS) abnormalities potentially associated with ZIKV infection and 23 had reported an increase in the incidence of the immune-mediated condition Guillain-Barré syndrome (GBS) or laboratory confirmed ZIKV in persons with GBS ( WHO situation report 10.03.2017). The causal association between ZIKV and adverse neurological outcomes has now been examined in many systematic and non-systematic reviews of research ^1, 2^. Case reports of other conditions in people with ZIKV infection, including immune-mediated idiopathic thrombocytopaenia purpura (ITP), have also been published ^3– 6^.

The World Health Organization (WHO) based its assessment, that there is “sufficient evidence to conclude that Zika virus is a cause of congenital abnormalities and is a trigger of GBS” ( WHO Zika causality statement), on a review of systematically identified studies up to May 30, 2016 and nonsystematically identified studies up to July 29, 2016 ^7^. The review addressed specific questions about 10 dimensions of causal associations, based on the work of Bradford Hill ^8^ and organised as a causality framework ( Supplementary Table 1) that covers: temporality (cause precedes effect); biological plausibility of proposed biological mechanisms; strength of association; exclusion of alternative explanations; cessation (reversal of an effect by experimental removal of, or observed decline in, the exposure); dose-response relationship; experimental evidence from animal studies; analogous cause-and-effect relationships found in other diseases; specificity of the effect; and the consistency of findings across different study types, populations and times. The review included 108 articles about congenital abnormalities or GBS but there was no, or insufficient evidence to answer questions in several dimensions of the causality framework ^7^. The causality framework included questions about ITP, but the review authors judged the number of published articles to be too low to assess causality. Since the WHO statement and accompanying publication, about 200 scientific publications every month are added to the body of evidence about all aspects of research about ZIKV.

A living systematic review would help to overcome some of the challenges of keeping up to date with the high volume of ZIKV research publications. A living systematic review is a systematic review that is “continually updated, incorporating relevant new evidence as it becomes available” ^9^, which can help in fields where evidence is emerging rapidly and where new review outcomes might change policy or practice decision ^10^. Technical solutions are available to facilitate the reviewing process, such as automated searching and deduplication and computer-assisted screening of article titles and abstracts, increase the efficiency and speed of a review team and transform the review into a living document.

This article aims to fulfil two separate objectives. First, we update our systematic review ^7^ with new evidence published from May 30, 2016 to January 18, 2017 about all 10 dimensions of the causal associations between ZIKV and (a) congenital brain abnormalities, including microcephaly, in the foetuses and offspring of pregnant women and (b) GBS/ITP in any population. Second, we describe the transition of the review into a living systematic review.

## Methods

### Classic protocol

We performed the review according to the protocol registered in PROSPERO CRD42016036693 ( PROSPERO protocol). The eligibility criteria, information sources and search strategy, study selection and data extraction are the same as reported in the protocol and in the previous publication ^7^. In brief, the search covers PubMed, Embase and LILACS electronic databases; the Pan American Health Organization (PAHO), WHO, the Centers for Disease Control and Prevention (CDC) and the European Centre for Disease Prevention and Control (ECDC) websites; and several preprint databases (BioRxiv, PeerJ and ArXiv). Search terms included ‘Zika virus’ and ‘ZIKV’ and corresponding MESH terms. Two reviewers screen and select articles for inclusion and extract data independently. We included publications that held information on at least one of the ten dimensions of the causality framework, regardless of the study design ^7^. We gathered publications systematically from May 30, 2016 to January 18, 2017 for this update. We refer to the previous version of the review as the baseline review ^7^ and to this current update as update 1. Reporting of the results follows the Preferred Reporting Items of Systematic reviews and Meta-Analyses (PRISMA) statement ( Supplementary File 1) ^11^.

### From systematic review to living systematic review

**Figure 1.**
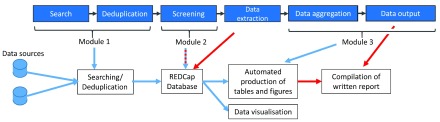
Living systematic review automation. Blue boxes and arrows represent the conceptual steps in a systematic review process. Automation is divided in three modules. Module 1 is the automation of the searching and deduplication of information from different data sources. Module 2 partly automates screening. Module 3 automates the production of tables and figures and outputs the data to a web platform (Data visualisation). Blue arrows represent automated information flows; red arrows represent manual input. The blue-red dashes arrow represents a blended form where reviewers verify automated decisions of the system. The white boxes show the practical implementation of the system and the data flow.

**Figure 2.**
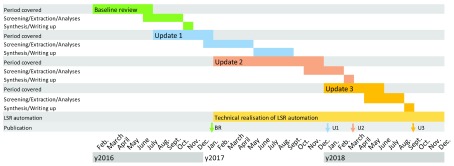
Timeline of review conduct, publication and transition to a living systematic review. The baseline review (BR, ^7^) and Update 1 (U1) this version classic, manual systematic review. During 2017 automation of the workflow was conducted resulting in a projected Update 2 (U2) and 3 (U3) with more rapid throughput. LSR, living systematic review.

To keep up with the quantity of published research, we developed a living systematic review workflow ( Supplementary File 2). We have identified three modules that could be automated ( Figure 1). As of December 2017, module 1, searching and deduplication, and part of module 3, the output of the report have been automated. Reviewers can be notified daily with a list of new unique search results so that screening can be performed rapidly. Following manual data extraction and synthesis, the output can be updated semi-automatically. We use the online database Research Electronic Data Capture (REDCap) ^12^ to maintain the references, perform screening and extract data into piloted extraction forms. We plan to update the review twice per year with formal peer reviewed updates ( Figure 2), and continually through a web platform.

We synthesised the findings as narrative summaries of the evidence according to causality dimension and outcome, as previously described ^7^, and compare them with the the baseline review. We use the term ‘confirmation’ to summarise findings of new studies included in update 1 if they report the same findings as those in the baseline review. We use the term ‘expansion’ of evidence if studies included in update 1 provide new findings.

## Results

**Figure 3.**
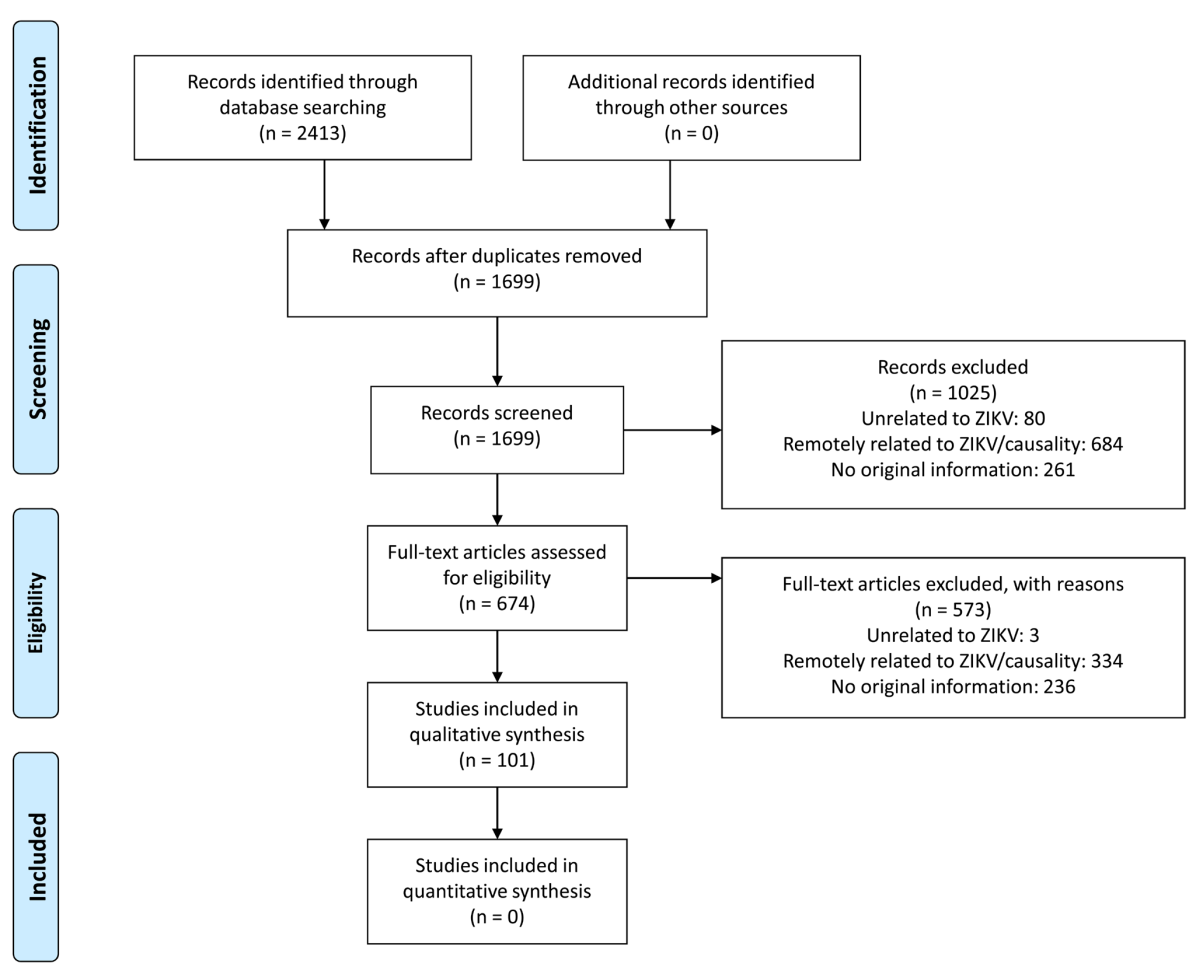
PRISMA flow diagram of included studies.

Between May 30, 2016 and January 18, 2017, we identified 2413 publications. After deduplication, we retained 1699 unique records. Based on screening of title and abstract, we discarded 1025 publications, retaining 674 items; after screening of the full text, 101 publications were included. Figure 3 shows the PRISMA flow diagram for this review ^11^. Seventy-seven publications held information on one or more dimensions of the causality framework on adverse congenital outcomes and 25 on GBS or idiopathic thrombocytopaenia purpura. Table 1 compares the included publications, study types and the causality dimension(s) they address in the baseline review ^7^ and update 1 of the review.

**Table 1.** Summary of included publications by study type and on which causality dimension they provide evidence. One publication can address multiple causality dimensions. Comparison between the current (U1) and the baseline review (BR, 7) stratified by outcome. GBS/ITP, adverse autoimmune outcomes (Guillain Barré syndrome/idiopathic thrombocytopaenia purpura). NA, not applicable; evidence about analogous conditions was not searched systematically; the dimension of consistency used information in items included for all other causality dimensions.

Condition and version	Adverse congenital outcomes		GBS/ITP	
	BR, N	U1, N	BR, N	U1, N
**Study type**				
Case report	9	13	9	5
Case series	22	12	5	11
Case-control study	0	3	1	1
Cohort study	1	8	0	0
Cross-sectional study	2	1	0	1
Controlled trials	0	0	0	0
Ecological study/outbreak report	5	4	19	7
Modelling study	2	0	0	0
Animal experiment	18	8	0	0
In vitro experiment	10	22	0	0
Sequencing and phylogenetics	3	3	2	0
Biochemical/protein structure studies	NA	3	NA	0
**Total:**	**72**	**77**	**36**	**25**
**Causality dimensions**				
Temporality	21	21	26	21
Biological plausibility	25	42	4	0
Strength of association	3	5	2	4
Alternative explanation	18	23	6	11
Cessation	2	0	6	2
Dose-response relationship	0	0	0	0
Experiment	20	11	0	0
Analogy	NA	NA	NA	NA
Specificity	0	1	0	0
Consistency	NA	NA	NA	NA

### Adverse congenital outcomes

A detailed overview of the new evidence is provided in Table 2 and Supplementary Table 2. In the search period for review update 1, an additional 548 cases of adverse congenital outcomes were described in 32 studies ^12– 43^. Adverse congenital outcomes described were: clinical microcephaly ^12– 17, 20– 24, 26– 31, 33, 35, 37, 40– 42^, imaging confirmed brain abnormalities ^12, 15, 17, 19– 24, 26– 31, 35, 37, 38, 40, 42^, intrauterine growth restriction ^15, 17, 31, 38, 40, 42^, ocular disorders ^12, 17, 27– 29, 31, 38, 40^ and auditory disorders ^12, 18, 29^.

**Table 2.** Summary of the evidence on the relation between ZIKV infection and adverse congenital outcomes. Evidence is displayed for each dimension and for each question of the causality framework. Zika virus (ZIKV); Dengue virus (DENV); West Nile virus (WNV); Chikungunya virus (CHIKV); Toxoplasmosis, Other [Syphilis, Varicella-zoster, Parvovirus B19], Rubella, Cytomegalovirus, and Herpes infections (TORCH); Central Nervous System (CNS). NA, not applicable; evidence about analogous conditions was not searched systematically; the dimension of consistency used information in items included for all other causality dimensions. the baseline review (BR), update 1 (U1).

Question	BR, N	U1, N	Summary
Temporality			
1.1a	18	19	Confirmation. Sufficient information to conclude that ZIKV infection precedes the development of congenital abnormalities in individuals ^12, 15– 18, 26– 31, 35– 38, 40, 42, 44, 45^.
1.1b	2	1	The peak of adverse congenital outcomes in Colombia was 24 weeks after infection ^45^ (similar to Brazil, 34 and 30 weeks ^7^).
1.2	18	19	Confirmation. Most mothers of infants with adverse outcomes were exposed to ZIKV during the first or the second trimester of their pregnancy ^34, 94^.Third trimester exposure can lead to brain malformations as well ^19^.
Biological plausibility			
2.1	1	6	Confirmation of the role of viral entry factors (receptor-ligand interaction) ^47– 52^.
2.2	1	4	Substantial expansion of the evidence on which cells express the receptors responsible for cell entry of ZIKV ^47, 50– 52^.
2.3	11	11	Expansion of evidence, sufficient information to conclude that ZIKV particles can be found in the umbilical cord blood and/or amniotic fluid of previously or currently infected mothers ^14, 23, 24, 32– 36, 38, 39, 42^.
2.4	0	7	The evidence that ZIKV particles found in tissue of the offspring are capable of replication was inconclusive in the previous version. In this update we found that *in vitro* evidence strongly indicates these ZIKV particles are capable of replication ^47, 50, 53– 55^. *Ex vivo* experiments demonstrate ZIKV capable of replication as well ^33, 36^.
2.5	6	7	Expansion of evidence, sufficient information to conclude that particles can be found in the brain and other tissues of cases with congenital abnormalities ^14, 17, 23, 24, 33, 34, 56^.
2.6	7	6	Confirmation. ZIKV particles found in the brain are capable of replication ^33, 56– 60^.
2.7	9	22	Strong expansion of evidence; Expansion of the understanding of how ZIKV causes congenital anomalies ^49, 52, 54, 57, 58, 60– 76^.
Strength of association			
3.1	2	5	Expansion of evidence on the strength of association at an individual level ^21, 22, 31, 40, 41^. However, the estimation of the effect size remains imprecise.
3.2	1	0	At a population level, confirmation lacks on the strength of association. However, 29 countries reported a relative increase in microcephaly cases during the ZIKV outbreak ( WHO situation report 05.01.2017).
Exclusion of alternatives			
4.1	18	23	Confirmation. In many epidemiological studies TORCH infections are assessed ^12, 14, 17– 19, 21– 28, 30, 31, 34, 36– 38, 40, 42, 45, 77^.
4.2	4	5	Confirmation. Exposure to toxic chemicals has been excluded ^12, 14, 18, 23, 28^.
4.3	0	0	No exclusion of alternative explanation: maternal/foetal malnutrition.
4.4	0	0	No exclusion of alternative explanation: hypoxic-ischaemic lesions.
4.5	3	7	Confirmation of evidence where the role of genetic conditions was excluded ^12, 18, 23, 28, 30, 36, 42^.
4.6	0	0	No exclusion of alternative explanation: radiation.
Cessation			
5.1	0	0	No publication with evidence that intentional removal of ZIKV infection in individuals leads to a reduction in congenital abnormalities.
5.2	0	0	No publication with evidence that intentional removal of ZIKV infection at population-level leads to a reduction of cases of congenital anomalies
5.3	2	0	Natural removal (end of epidemic) leads to a reduction in microcephaly cases in Brazil; Other countries have shown a decrease in reported microcephaly cases as the cumulative ZIKV incidence plateaued ( http://www. paho.org/hq/index.php?option=com_content&view=article&id=12390&Itemid=42090&lang=en).
Dose-response			
6.1	0	0	No publication with evidence that the risk of adverse congenital outcomes is associated with the viral load in the mother.
6.2	0	0	No publication with evidence that the clinical severity of the infection of the mother determines the severity of the congenital anomalies. In one cohort study, symptoms in the mother did not influence the outcome ^32^.
Animal experiments			
7.1	3	3	Expansion of the evidence that the inoculation of pregnant female animals (mice and macaques) with ZIKV causes congenital anomalies in the offspring ^78, 84, 85^.
7.2	10	3	Confirmation of the evidence that the intracerebral inoculation of newborn mice with ZIKV leads to ZIKV replication in the CNS ^81, 82, 86^.
7.3	8	3	Expansion of the evidence that other routes of inoculation of newborn animals with ZIKV leads to ZIKV replication in the CNS (intravaginal infection of adult mice, subcutaneous infection of newborn mice) ^79, 80, 84^.
7.4	1	8	Expansion of the evidence that other experiments with animals or animal-derived cells support the association of ZIKV infection and congenital anomalies ^63, 71, 78– 83^.
Analogy			
8.1	NA	NA	CHIKV was shown to be vertically transmissible and lead to adverse congenital outcomes ^88^.
8.2	NA	NA	Confirmation. Congenital ZIKV analogous to other TORCH infections ^87^.
8.3	NA	NA	For most analogous pathogens, infections earlier in the pregnancy have a higher risk of adverse outcomes.
Specificity			
9.1	0	1	Expansion of evidence for distinct congenital Zika syndrome. Unique pattern of five features suggested: severe microcephaly with overlapping cranial structures, subcortical location of brain calcifications, macular scarring and retinal mottling, congenital contractures and early pyramidal and extrapyramidal symptoms ^89^.
Consistency			
10.1	NA	NA	Confirmation. ZIKV-related adverse congenital outcomes in different regions (South America, Central America, and the Pacific region). The proportion of cases varies over geographic regions/time.
10.2	NA	NA	Confirmation. ZIKV exposure and adverse congenital outcome in different populations (people living in ZIKV endemic areas and travellers.
10.3	NA	NA	No publication with evidence of consistency across lineages due to circulation of single strain.
10.4	NA	NA	Confirmation. ZIKV exposure and adverse congenital outcomes found in different study types.

**Temporality.** This update confirms the previous conclusion that ZIKV infection precedes the adverse congenital outcomes. We found an additional 21 publications in which ZIKV infection preceded the adverse congenital outcome at an individual level ^12, 15– 18, 26- 31, 35- 40, 42, 44, 45^ and at a population level ^45, 46^. Infections in the first and second trimester seemed to be related to the most adverse outcomes ^31, 40^. Cohort studies of pregnant women from French Guiana and Brazil found a higher proportion of congenital abnormalities in babies born from mothers infected in the first and the second trimester ^31, 40^.

**Biological plausibility.** This update includes an additional 42 studies ^14, 17, 23, 24, 32– 36, 38, 39, 42, 47– 76^, some of which expand the evidence base. Whereas in the baseline review, we found inconclusive evidence of whether ZIKV particles in infants were capable of replication, both *in vivo* and *ex vivo* studies now demonstrate that this is the case ^33, 36, 47, 50, 53- 55^. Furthermore, there was a strong expansion of the evidence clarifying how ZIKV causes adverse congenital outcomes. ZIKV uses receptors from the TAM family to enter cells ^47– 52^, where the virus induces cell death, primarily in developing neuronal cells ^60, 61, 64, 65, 67, 69, 70, 75^.

**Strength of association.** We included five publications that confirm a strong association between ZIKV infection and adverse congenital outcomes ^21, 22, 31, 40, 41^. The strength of association at an individual level was high but imprecise, owing to small sample sizes. Estimates from cohort studies ^31, 40^ appeared to be lower than those from case-control studies ^21, 22, 41^. The definition of the outcomes and the outcomes assessed, varied between studies. The risk of any adverse congenital outcomes was higher and more variable than the risk of microcephaly. The risk ratio for microcephaly between ZIKV unexposed and exposed was 4.4 (95% CI: 0.2-80.8) in a cohort in Brazil ^31^ and 6.6 (95% CI: 0.8-56.4) in a cohort in French Guiana ^40^. In the Brazilian cohort ^31^, the proportion of any adverse congenital outcomes among ZIKV infected women was high (41.9% [49/117]), compared with the uninfected group (5.2% [3/57]). In a prospective case- control study in Brazil, women with laboratory-confirmed ZIKV had 55.5 (95% CI: 8.6-infinity) times the odds of having a baby with microcephaly compared with women without evidence of ZIKV infection ^21^. A retrospective case- control study in Hawaii found an odds ratio of 11.0 (95% CI: 0.8-147.9) ^41^. In the latter, however, exposure was assessed retrospectively using serology.

**Exclusion of alternatives.** We included 23 new studies in this update ^12, 14, 17– 19, 21– 28, 30, 31, 34, 36– 38, 40, 42, 45, 77^. Many studies included in this review that reported on adverse outcomes of congenital ZIKV excluded TORCH infections ^12, 14, 17– 19, 21– 28, 30, 31, 34, 36– 38, 40, 42, 45, 77^; exposure to toxic chemicals ^12, 14, 18, 23, 28^ or genetic conditions ^12, 18, 23, 28, 30, 36, 42^. Maternal or foetal malnutrition, hypoxic-ischaemic lesions and underlying genetic conditions were not excluded. No single alternative explanation could be given to explain the relation between ZIKV and adverse congenital outcomes.

**Cessation.** We did not find any new publications for this causality dimension. Evidence is still lacking on the effect of intentional removal due to lack of vaccination or elimination of mosquitoes on a large scale.

**Dose-response.** There is still no direct evidence about the association between Zika viral load and probability of adverse congenital outcome in observational studies, or of an association between symptomatic status and outcome. In a study in the United States, Honein *et al.* found similar proportions of adverse congenital outcomes in symptomatic and asymptomatic ZIKV-infected mothers ^32^.

**Animal experiments.** This update of the review includes an additional 11 studies ^63, 71, 78– 86^. These studies confirm a consistent relation between a range of contemporary ZIKV and adverse congenital outcomes, including from Brazil ^85^, Puerto Rico ^79^ and Mexico ^80, 81^. The body of evidence coming from animal studies has grown; both in mice and macaques, congenital anomalies such as intra-uterine growth restriction and signs of microcephaly were observed after ZIKV infection ^78, 84, 85^.

**Analogy.** As for the baseline review, evidence for this dimension was not reviewed systematically because our search strategy did not include terms for other infections or conditions. Studies included in this version of the review confirm the analogy between congenital ZIKV and TORCH infections ^87^. Vertical transmission of West Nile virus and dengue virus were summarised in the baseline review. In update 1, we included a case series from El Salvador that reported Chikungunya virus in 169 newborns of women with symptomatic infection; a minority had CNS infection, but microcephaly was not reported ^88^. For most analogous pathogens, infections earlier in the pregnancy have a higher risk of adverse outcomes ^87^.

**Specificity.** We included one study ^89^, suggesting an expansion of evidence of a distinct congenital Zika syndrome (CZS) ^89^. In a review of 34 published reports, the authors suggest five congenital abnormalities that, in conjunction, comprise a pattern that is unique to ZIKV: severe microcephaly with overlapping cranial structures, subcortical location of brain calcifications, macular scarring and retinal mottling, congenital contractures and early pyramidal and extrapyramidal symptoms ^89^.

**Consistency.** The studies included in this version of the review confirm the pattern of consistency observed in the baseline review. ZIKV infection in association with adverse congenital outcomes were reported in a range of study designs from different regions ( WHO situation report 05.01.2017), although the proportion of affected infants varies over geographic region and time. ZIKV exposure resulted in adverse congenital outcome in people living in ZIKV endemic areas ^12– 19, 21– 34, 40– 42, 44, 45, 77, 90, 91^ and in female travellers who returned to non-endemic countries ^34– 39, 92, 93^. Direct evidence from epidemiological studies comparing different lineages is lacking due to circulation of a single strain.

**Conclusion.** The evidence added in update 1 of the review confirms the conclusion of a causal association between ZIKV and adverse congenital outcomes. New findings expand the evidence base in the dimensions of biological plausibility, strength of association, animal experiments and specificity. *In vitro* and *in vivo* studies elucidate pathways on how these outcomes likely occur. Conclusive evidence on the strength of association is lacking. Studies provide crude overall measures of association, not taking into account potential co-factors.

### GBS/ITP

In the search period for update 1 of the review, an additional 154 cases of ZIKV-related GBS ^95^ and 11 ZIKV-related cases of ITP ^3– 6^ were described in 18 studies. Table 3 summarises the evidence for specific questions in each of 10 causality dimensions (detailed overview in Supplementary Table 3).

**Table 3.** Summary of the evidence on the relation between ZIKV infection and adverse autoimmune outcomes. Evidence is displayed for each dimension of the causality framework and for each question. Zika virus (ZIKV); Dengue virus (DENV); Guillain-Barré syndrome (GBS); immune-mediated idiopathic thrombocytopaenia purpura (ITP). NA, not applicable; evidence about analogous conditions was not searched systematically; the dimension of consistency used information in items included for all other causality dimensions. the baseline review (BR), Update 1 (U1).

Question	BR, N	U1, N	Summary
Temporality			
1.1a	9	17	Expansion of the evidence. Additional case reports and case series were identified that confirmed that ZIKV infection preceded adverse autoimmune outcomes ^3, 5, 6, 95– 102, 103– 108^.
1.1b	9	4	Expansion of the evidence that on the population level ZIKV precedes GBS or ITP ^103, 109– 111^.
1.2	7	14	Expansion of evidence that the interval between exposure to ZIKV and occurrence of symptoms is typical for para- or post-infectious autoimmune-mediated disorders ^5, 6, 95– 102, 103– 106, 112^.
Biological plausibility			
2.1	3	0	No additional evidence was identified that ZIKV epitopes mimic host antigens (molecular mimicry).
2.2	1	0	No additional evidence was identified that ZIKV infection leads to an increased in detectable autoreactive immune cells or autoreactive antibodies.
2.3	0	0	There is no evidence on other biologically plausible mechanisms of ZIKV infection leading to GBS/ITP.
Strength of association			
3.1	1	0	No additional evidence was identified on the association between Zika infection and GBS/ITP at the individual level.
3.2	2	4	Expansion of evidence. GBS incidence increased in several regions, during the same time ZIKV was circulating ^103, 109– 111^.
Exclusion of alternatives			
4.1	7	9	Confirmation of the evidence where other infections were assessed. However, often previous DENV infection was reported, and not excluded ^4– 6, 95, 98, 101, 103, 104, 111^.
4.2	0	1	Expansion on the evidence where vaccines were excluded ^5^.
4.3	0	5	Expansion on the evidence where other systemic illnesses were excluded ^4– 6, 95, 99, 112^.
4.4	0	2	Expansion on the evidence where medication, drugs or other chemicals was excluded ^99, 112^.
Cessation			
5.1	0	0	No relevant studies identified that intentional removal or prevention of ZIKV infection in individuals leads to a reduction in cases with GBS/ITP.
5.2	0	0	No relevant studies identified that intentional removal or prevention of ZIKV infection at population level leads to a reduction in cases with GBS/ITP.
5.3	6	2	Expansion. Additionally, in Venezuela and the Dominican Republic, it was shown that GBS cases decreased with a decrease in reported ZIKV cases ^103, 111^.
Dose-response			
6.1	0	0	No relevant studies identified that the risk and the clinical severity of GBS/ITP are associated with viral titres.
Animal experiments			
7.1	0	0	No relevant studies identified where the inoculation of animals with ZIKV leads to an autoimmune reaction resulting in peripheral neuropathy or thrombocytopenia.
7.2	0	0	No relevant studies identified that other animal experiments support the association of ZIKV infection and GBS/ITP.
Analogy			
8.1	NA	NA	No additional studies identified that other flaviviruses or arboviruses cause GBS/ITP.
8.2	NA	NA	No additional studies identified that other pathogens cause GBS/ITP.
8.3	NA	NA	No additional studies identified that explain which pathogen or host factors facilitate the development of GBS/ITP.
Specificity			
9.1	0	0	No relevant studies identified that pathological findings in cases with GBS/ITP are specific for ZIKV infection.
Question	v1, N	v2, N	Summary
Consistency			
10.1	NA	NA	Confirmation that the association between ZIKV cases and cases with GBS is consistently found across different geographical regions.
10.2	NA	NA	Confirmation that the association between ZIKV cases and cases with GBS is consistently found across different populations/subpopulations.
10.3	NA	NA	No additional studies identified that the association between ZIKV cases and cases with GBS/ITP is consistently found across different ZIKV lineages/strains.
10.4	NA	NA	Confirmation that the association between ZIKV cases and cases with GBS is consistently found across different study designs.

**Temporality.** We found an additional 17 publications that confirmed that ZIKV infection preceded the GBS or ITP at an individual level ^3, 5, 6, 95– 108^ or at a population level ^103, 109– 111^. ZIKV infections seems to be followed by GBS on average between 5 and 10 days. In one case series from Colombia ^103^, the authors distinguished between rapid onset of GBS symptoms after ZIKV symptoms (para-infectious) and post-infectious onset, with an asymptomatic period after ZIKV symptoms before the start of GBS symptoms.

**Biological plausibility.** We did not find any publications about the biological plausibility of ZIKV as a cause of GBS or ITP.

**Strength of association.** We did not find any comparative observational studies during the search period for update 1. Several surveillance studies confirmed an increase in notified GBS cases during ZIKV outbreaks at the population level ^111^. Rate ratios were significantly higher for Brazil, Colombia, the Dominican Republic, El Salvador, Honduras, Suriname and Venezuela when comparing pre-ZIKV GBS incidence and the incidence during the outbreak ^111^; this ratio ranged from 2.0 (95% CI: 1.6-2.6) to 9.8 (95% CI: 7.6-12.5).

**Exclusion of alternatives.** We included 11 publications ^4– 6, 95, 98, 99, 101, 103, 104, 111, 112^ that expanded the list of alternative causes for autoimmune disease that were excluded, such as infections, vaccines, other system illnesses and medication, drugs or other chemicals. Many GBS cases in these publications had serological evidence of previous exposure to DENV, as seen in the baseline review. It remains unclear how large the potential role of co-factors such as antibody dependent enhancement are.

**Cessation.** We did not identify any publications with evidence about the effect of intentional removal/elimination/prevention of ZIKV on either GBS or ITP. An additional publication confirmed evidence that the natural removal of ZIKV resulted in a decrease in GBS cases in Brazil, Colombia, Dominican Republic, El Salvador, Honduras, Suriname and Venezuela ^104, 111^.

**Dose-response.** We did not identify any publications about this dimension for either GBS or ITP.

**Animal experiments.** No additional evidence from animal experiments was identified that support the association between ZIKV infection and GBS/ITP development.

**Analogy.** As for the baseline review, evidence for this dimension was not reviewed systematically because our search strategy did not include terms for other infections or conditions. We did not identify any new publications addressing this dimension for either GBS or ITP.

**Specificity.** We did not identify any new publications addressing this dimension for either GBS or ITP.

**Consistency.** Studies included in update 1 confirmed the consistency of the evidence for 3 of 4 questions about the association between ZIKV and GBS. By geographical region, ZIKV transmission has been associated with the occurrence of GBS in 2 of 4 regions; increased GBS incidence has been reported in the WHO regions of the Americas and the Western Pacific region, but not in the African or Southeast Asian region, despite recent ZIKV circulation ^113^. By study design, the association between ZIKV infection and GBS has been found at individual and population level and with different study designs. By population, ZIKV infection has been linked to GBS in ZIKV endemic regions ^4– 6, 95, 96, 98– 101, 103– 105, 109, 111, 114^ and travellers from non-affected countries who were exposed in these endemic regions ^3, 97, 102, 106, 112^. There was insufficient evidence to examine the consistency of evidence about ZIKV and ITP.

**Conclusion.** The body of evidence has grown during the search period for update 1 but only for dimensions that were already populated in the original publication for GBS. There is still a limited understanding of the biological pathways that potentially cause the occurrence of autoimmune disease following ZIKV infection. Additionally, prospective comparative epidemiological studies are still lacking. It remains unclear how co-factors such as age and previous exposure to flaviviruses influences the risk of developing GBS. The evidence supports a temporal association between ZIKV and ITP but there is an absence of evidence for other dimensions of causality.

### Search results from January 19, 2017 to January 05, 2018

Automated search and deduplication processes identified 2410 publications about any aspect of ZIKV infection. The next update of this review will address causality dimensions in the realm of epidemiological studies; strength of association, dose-response relationship, specificity and consistency.

## Discussion

**Statement of principal findings.** This systematic review confirms evidence of a causal association between ZIKV and adverse congenital outcomes and between ZIKV and GBS, although evidence about biological plausibility is still lacking. We assessed evidence about an association between ZIKV and ITP but found that this only addressed the dimension of temporality. The review is transitioning from classic systematic review methods to those of a living systematic review.

**Strengths and limitations of the study.** The strengths of this study are the systematic approach to the identification, selection and extraction of data following a causality framework that provides a structure for the consideration of heterogeneous sources of evidence and a large set of review questions. Automation of the review output allows rapid updating of tables of results. We have also developed methods to automate search and deduplication of search results to make the transition to a living systematic review that will allow continual updating of results. The main limitation of the classic systematic review of such a complex topic is the high workload and time required to maintain it. Another limitation, resulting from the large number of review questions, is the time taken to resolve inter-reviewer differences in interpretation of eligibility criteria. This could have resulted in subjectivity over decisions about inclusion in the review. Although a second reviewer checked all extractions, changes in the review team could introduce inconsistency. As in the baseline review, we used case definitions as authors described them in individual publications. This potential source of information bias is likely to decrease over time as standardised case definitions and protocols are adopted ^115^. As in the previous version, we did not systematically apply quality assessment tools to individual studies. Because much of the technical infrastructure was built as the evidence emerged, output was delayed. As much of the LSR methodology was novel, it took time to find a balance between speed and efficiency.

**Strengths and weaknesses in relation to other publications.** Our systematic review differs from most standard reviews because of the number of questions within the dimensions of the causality framework and the number of outcomes. Other recent examples of living systematic reviews only distinguish between two study types (RCT and non-RCT) ^116^ and are guided by only a small set of review questions ^117, 118^. Our review conclusion, confirming evidence for a causal association between ZIKV and GBS differs from that of a review ^119^ of the findings of four case reports ^104, 120– 122^ and one case-control study ^123^. The authors found insufficient evidence to confirm the presence of an acute motor axonal neuropathy variant of GBS. They did not, however, suggest an alternative explanation for the increase in incidence of GBS in the countries that experienced ZIKV outbreaks. The two versions of our review included 64 publications about ZIKV and GBS across ten dimensions of causality.

**Meaning of the study: possible mechanisms and implications for basic researchers, clinicians or policymakers.** The conclusions on the causal relation between ZIKV and adverse congenital outcomes and ZIKV and GBS did not change with this update. We found insufficient evidence about the association between ZIKV and ITP to state with certainty that there is a causal association. The total volume of evidence about the association between ZIKV and GBS is less than for the association with adverse congenital outcomes. There is, in particular a lack of published research to elucidate biological mechanisms for direct neuronal or autoimmune damage in GBS ^124^. The descriptive data about the numbers and types of different studies over time illustrates how evidence about a new, or re-emerging, infection emerges over time. The evidence from many regions that were affected by the ZIKV outbreak remains limited to anecdotal evidence of adverse outcomes, in the form of case reports or case series. The slowing of ZIKV transmission in 2017 means that fewer people are being affected by ZIKV and its complications and fewer people are being enrolled into prospective studies. Further progress in epidemiological research will rely more heavily on research consortia who are contributing to joint analyses of data from existing studies.

**Unanswered questions and future research.** As the volume and complexity of the evidence in different causality dimensions accumulates, the need for expert input and interpretation of the findings of this systematic review increases. The focus of research on ZIKV and causal associations with different types of adverse outcomes is also changing. For congenital abnormalities resulting from ZIKV vertical transmission, epidemiological research should examine CZS in comparative studies, quantify the strength of association with ZIKV, clarify associations with gestational age, symptomatology and viral load and further investigate potential co-factors such as previous dengue infection and flavivirus vaccination. WHO standardised study protocols provide suggestions for exclusion of alternative explanations and exploration of co-factors ( Harmonization of ZIKV Research Protocols). For GBS, epidemiological studies are needed to quantify the association with ZIKV more precisely, but also to determine whether there are distinct phenotypes resulting from autoimmune mechanisms or direct neuronal involvement. For ITP, additional evidence across all causality dimensions is needed.

**Planned updates of a living systematic review.** Living systematic review methodology and techniques will continue to develop. Since a chain is only as strong as its weakest link, any processing step has the potential to slow down a living systematic review. Clearly defined protocols that define update frequencies and throughput speed of different actors in the publishing process are vital. The next update of the systematic reviews will use living systematic review methods to assess the evidence for 2017 and early 2018 (update 2, Figure 2). The review will, for the first time, separate evidence from epidemiological study designs from *in vitro* and *in vivo* laboratory studies. We will narrow down the inclusion criteria based on study type. Epidemiological evidence will address the causality dimensions ‘strength of association’, ‘dose-response’, ‘specificity’ and ‘consistency’. Several co-factors might play a role in the strength of association. Thus, we will continue to collect information on previous dengue virus infection, yellow fever vaccination status, socioeconomic status, gestational age and others factors that might play a role in the severity of the outcome. We will amend the protocol with a more focused search strategy and inclusion criteria ( Supplementary File 3).

Systematic reviews of questions addressed by laboratory studies are less frequent than those addressing epidemiological research questions. There is still need to update understanding of the causality dimensions ‘biological plausibility’ and ‘animal experiments’, particularly to increase our understanding of biological pathways for ZIKV effects on the peripheral nervous system and the immune system. We encourage and welcome collaboration from scientists with expertise in these fields to update systematic reviews for these causality dimensions.

**Conclusion.** This systematic review confirms previous conclusions that ZIKV is a cause of congenital abnormalities, including microcephaly and is a trigger of GBS. Evidence suggests an association with idiopathic thrombocytopaenia purpura but is not conclusive. The transition to living systematic review techniques and methodology provides a proof of concept for the use of these methods to synthesise evidence about an emerging pathogen such as ZIKV, ultimately leading to integration in the whole public health information cycle ^125^. With the infrastructure for living systematic review methods and open source access to the software and outputs, we aim to enhance outbreak preparedness and the study of emerging and re-emerging pathogens.

## Data availability

All data underlying the results are available as part of the article and no additional source data are required.

## Supplementary material

Supplementary Table 1 – Bradford Hill’s “viewpoints” of causation.

Supplementary Table 2 – Evidence table adverse congenital outcomes update 1.

Supplementary Table 3 – Evidence table GBS/ITP update 1.

Supplementary File 1 – PRISMA Checklist.

Supplementary File 2 – LSR automation methodology.

Supplementary File 3 – Search strategy update 2.
