# Supplementary material for: Zika virus infection as a cause of congenital brain abnormalities and Guillain-Barré syndrome: From systematic review to living systematic review
Source: F1000Res. 2018 Feb 15;7:196. [Version 1] doi: 10.12688/f1000research.13704.1 (PMC6290976; doi:10.12688/f1000research.13704.1)
Supplement: Supplementary file 1 [file f1000research-7-14886-s0000.tgz › 95380c74-7569-4049-bf3b-b2832794bdf9.docx]

# **Supplementary Table 1 – Bradford Hill’s “viewpoints” of causation**

Originally published as supplement to [1] available from: <https://doi.org/10.1371/journal.pmed.1002203.s004>

Supplementary Table 1. Bradford Hill’s “viewpoints” of causation, modifications by Gordis and adaptation to the dimensions of the causality framework, in order

| **Order** | **Bradford Hill viewpoints^a^** [2] | **Gordis guidelines I^b^** [3] | **Gordis guidelines II^c^** [3] | **Zika causality framework dimensions** | **Explanation of modifications to the Bradford Hill and Gordis lists** |
| --- | --- | --- | --- | --- | --- |
| 1 | Strength (listed 2^nd^ in [4]) | Temporal relationship | Temporal relationship (major) | Temporality | Same as Gordis I and II |
| 2 | Consistency of observed association (listed 1^st^ in [4]) | Strength of the association | Biological plausibility (major) | Biologic plausibility | Same as Gordis I and II |
| 3 | Specificity (same rank as in [4]) | Dose-response relationship | Consistency (major) | Strength of association | Strength of association moved up because of the importance of comparing exposed and non-exposed populations. Consistency moved to the end because its assessment summarises evidence from studies that have addressed the other dimensions. |
| 4 | Temporality (same rank as in [4]) | Replication of the findings (maps onto consistency in Bradford Hill’s list) | Alternative explanations (major) | Exclusion of alternate explanations | Same as Gordis I and II |
| 5 | Biological gradient | Biologic plausibility | Dose-response relationship (other) | Cessation | See above |
| 6 | Plausibility | Consideration of alternate explanations (not in Bradford Hill’s list) | Strength of the association (other) | Dose-response relationship | Dose-response relationship moved down because of a priori assumption that data would be scarece |
| 7 | Coherence (listed 5^th^ in [4]) | Cessation of exposure (maps onto experiment in Bradford Hill’s list) | Cessation effects (other) | Animal experiments | This dimension allows “experiment” to mean either laboratory experiments or clinical trials [5] |
| 8 | Experiment | Consistency with other knowledge (maps onto coherence in Bradford Hill’s list) |  | Analogy | Taken from Bradford Hill’s list |
| 9 | Analogy | Specificity |  | Specificity | Taken from Bradford Hill’s list |
| 10 |  |  |  | Consistency of the association |  |

1. Bradford Hill’s list and relationship to the criteria used by the advisory group to the US Surgeon General’s report on smoking and health [4];
2. Gordis guidelines I are the adaptation of items used by Bradford Hill [2] and the US Surgeon General’s report on smoking and health [4];
3. Gordis guidelines II are the items in the US Public Health Service modified list to evaluate evidence of a causal relationship (cited in [3]). The items are split into major criteria and other considerations.

This table has been reproduced with permission from [Krauer F, Riesen M, Reveiz L, Oladapo OT, Martinez-Vega R, Porgo TV, et al. Zika Virus Infection as a Cause of Congenital Brain Abnormalities and Guillain-Barre Syndrome: Systematic Review. PLoS Med. 2017;14(1):e1002203. doi: 10.1371/journal.pmed.1002203. PubMed PMID: 28045901; PubMed Central PMCID: PMC5207634.]

## References

1. Krauer F, Riesen M, Reveiz L, Oladapo OT, Martinez-Vega R, et al. (2017) Zika Virus Infection as a Cause of Congenital Brain Abnormalities and Guillain-Barre Syndrome: Systematic Review. PLoS Med 14: e1002203.

2. Hill AB (1965) The Environment and Disease: Association or Causation? Proc R Soc Med 58: 295-300.

3. Gordis L (2009) Chapter 14. From Association to Causation: Deriving Inferences from Epidemiologic Studies. Epidemiology: Saunders Elsevier. pp. 227-246.

4. US Department of Health E, and Welfare. Public Health Service, (1964) Smoking and Health. Report of the Advisory Committee to the Surgeon General of the Public Health Service. Public Health Service Publication No. 1103. Washington DC. 1-386 p.

5. Rothman KJ, Greenland S (2005) Causation and causal inference in epidemiology. Am J Public Health 95 Suppl 1: S144-150.
