# Supplementary material for: Zika virus infection as a cause of congenital brain abnormalities and Guillain-Barré syndrome: From systematic review to living systematic review
Source: F1000Res. 2018 Feb 15;7:196. [Version 1] doi: 10.12688/f1000research.13704.1 (PMC6290976; doi:10.12688/f1000research.13704.1)
Supplement: Supplementary file 2 [file f1000research-7-14886-s0001.tgz › 1f984c8c-be73-4c3a-b1da-f0959763754c.docx]

# Supplementary Table 2 – Evidence table adverse congenital outcomes update 1

| **Question No** | **Causality dimension + question** | **Study type (number) references** | **Country of study** | **Support of causality dimension** | **Evidence against causality** | **Summary** |
| --- | --- | --- | --- | --- | --- | --- |
| **Temporality** | | | | | | |
| **1.1a** | Does Zika infection precede the development of congenital anomalies? | Case series (4): [1-4] Case report (8): [5-12] Cohort study (6): [13-18] Ecological study/outbreak report (1): [19] | United States, Martinique, Brazil, Suriname, Colombia, French Guiana, Slovenia | 19 | 1 | Eight case reports describe mother-infant pairs with infants with adverse congenital outcomes. ZIKV infection preceded the described adverse outcomes [4-10]. Five case series describe 2-40 mother-infant pairs where in at least one pair ZIKV was diagnosed before the adverse congenital outcome [1-4, 11]. In six cohort studies, ZIKV infection in the mother preceded the adverse congenital outcome in the infant [13-18]. Temporal trends in a surveillance report from Colombia showed an increase in ZIKV followed by an increase in adverse congenital outcomes [19]. [20] describes two mothers exposed in 2nd trimester that did not lead to adverse congenital outcomes, despite the presence of ZIKV in the amniotic fluid of one woman. |
| **1.1b** | Is there a consistent time-dependent relationship between the occurrence of Zika cases and cases with congenital anomalies at population-level? | Ecological study/outbreak report (1): [19] | Colombia | 1 | 1 | A surveillance report from Colombia [19] showed that the peak of microcephaly occurred approximately 24 weeks after the peak in ZIKV cases. A surveillance report from Brazil [21] showed a decrease of microcephaly incidence for the whole country despite an increase of ZIKV cases. |
| **1.2** | Is the timing of Zika infection during gestation and the observed pattern of congenital anomalies compatible with the expected stage of embryological development? | Case report (8): [5, 7-12, 22] Case series (4): [1-4] Cohort study (6): [13-18] In vitro experiment (1): [23] | Spain, United States, Martinique, Brazil, Colombia, French Guiana, Slovenia | 19 | 2 | Most mothers with infants with adverse outcomes were exposed to ZIKV during the first or the second trimester of their pregnancy [1-5, 7-18, 22]. However, [24] describes two cases of ZIKV infection in the 36th week of pregnancy whose foetuses had preserved head circumference at birth and findings of sub ependymal cysts and lenticulostriate vasculopathy in postnatal imaging. [23] demonstrates that first-trimester human maternal-decidual tissues grown ex vivo as organ cultures. An efficient viral spread in the decidual tissues was demonstrated. Furthermore, efficient spread was demonstrated in maternal decidual tissue midgestation. [20] describes two women with third trimester exposure that did not lead to brain abnormalities, although in one infant ZIKV was demonstrated. |
| **Biological plausibility** | | | | | | |
| **2.1** | Which cell receptor(s) bind the ligand of ZIKV in humans? | In vitro experiment (5): [25-29] Sequencing and phylogenetics (1): [30] | Uganda, Nicaragua | 6 | 0 | Five in vitro experiment demonstrate that ZIKV is using viral entry factors from the TAM family (AXL, Mer and Tyro3), mainly AXL to enter different cell types [25-29]. Sensistivity of different tissues might depend on expression of AXL [27, 29]. The ZIKV protein NS1 functions in genome replication and host immune-system modulation. Because the NS1 protein from WNV and DENV2 interacts with parts of the complement system and Toll like receptors, the authors focus on the evolution of structural proteins. modulate some aspect of viral fitness, [30] |
| **2.2** | Which tissues express such receptor(s) and at which gestational age are they expressed? | In vitro experiment (4): [25, 27-29] | Uganda, Nicaragua | 4 | 0 | [25] demonstrates that several primary cell types (of the human placenta isolated from mid- and late-gestation- cytotrophoblasts, endothelial cells, fibroblasts, and Hofbauer cells in chorionic villi and amniotic epithelial cells and trophoblast progenitors in amniochorionic membranes) and explants from the human placenta express AXL, Tyro3 and TIM1. In [27] freshly isolated cytotrophoblasts from first and second trimester placentas were demonstrated to be susceptible to ZIKV infection. In tissue samples at peak neurogenesis (13-16 weeks post conception) ZIKV preferred glial cells. After 18 weeks post conception, infection was seen in astrocytes, likely due to high expression of AXL; blocking of AXL reduces infection. Neurons were less susceptible to infection. [28] confirmes that astrocytes and glial cells are more susceptible than neurons. [29] demonstrates that both the African and the Asian strain infect human umbilical vein endothelial cells. They demonstrate that AXL is required for ZIKV entry post binding; this suggests a role for haematogenous dissemination. |
| **2.3** | Can ZIKV particles be found in the placenta, umbilical cord blood and/or amniotic fluid of previously or currently infected mothers and if yes, with what probability? | Case report (4): [5, 6, 11, 22] Case series (6): [1, 20, 31-34] Cross-sectional study (1): [35] | Spain, Brazil, United States, Martinique, Suriname, Barbados, Belize, Colombia; Dominican Republic, El Salvador, Guatemala, Haiti, Honduras; Mexico, Republic of Marshall Islands, Venezuela | 11 | 0 | Eleven observational studies (four case reports, six case series and one cross-sectional study) showed the presence of viral particles in the placenta, umbilical cord blood and/or amniotic fluid [1, 5, 6, 11, 20, 22, 31-35]. A cross-sectional study, in a group of women from the USA with evidence of possible ZIKV, demonstrated the presence of virus in 10 placentas out of 26 infants with congenital abnormalities using RT-PCR [35]. |
| **2.4** | Are the ZIKV particles in the placenta/amniotic fluid/umbilical cord infectious/capable of replication? | In vitro experiment (5): [25, 27, 36-38] Case report (1): [6] Case series (1): [34] | Uganda, Nicaragua, United States, Columbia, Brazil, Suriname, France | 7 | 0 | [25, 36-38] demonstrate that viral replication in vitro is possible in placental cells and human trophoblasts. [6, 34] used in situ hybridization to demonstrate the presence of ZIKV in placental tissue; in the amniotic epithelium and in foetal mesenchymal cells, more specific in the perichondrium. |
| **2.5** | Can ZIKV particles be found in brain (or other) tissues of cases with congenital anomalies? | In vitro experiment (1): [39] Case report (1): [22] Case series (5): [3, 31-34] | Spain, Brazil, Puerto Rico, United States, Columbia, Slovenia | 7 | 3 | One case report and five case series reported ZIKV particles diagnosed using RT-PCR in the brain or other tissues of infants with adverse congenital outcomes [3, 22, 32-34]. One in vitro study demonstrated replication in human neuronal progenitor cells. On the contrary, several case reports and case series failed to demonstrate ZIKV in the tissues of infants with adverse congenital outcomes [1, 9]. [40] discovered no ZIKV peptides in protein extracts of three Zika positive brains, using mass spectrometry. However, bovine viral diarrhoea virus-like peptides were discovered, leading to the speculative hypothesis that other etiological agents might play a role. |
| **2.6** | Are the ZIKV particles found in the brain infectious/capable of replication? | In vitro experiment (5): [39, 41-44] Case series (1): [34] | United States, Brazil, Columbia | 6 | 0 | [42] show that ZIKV is capable of replication in neural stem cells derived from induced pluripotent cells in vitro. [41] shows the effective infection of human foetal neural stem cells. [39] showed that human neural progenitor cells were susceptible to ZIKV and that the virus persisted up to 28 days in these cells in vitro. [34] showed that in infants with microcephaly ZIKV replicates and persists in the brain. [43] demonstrate that cranial neural crest cells are susceptible to infection with ZIKV, but not the DENV. ZIKV infected cranial neural crest cells undergo limited apoptosis but these cells mitigate the cells death and aberrant differentiation of neural progenitor cells. [44] show the presence of ZIKV particles in the cytoplasm of astrocytes and neurons of organoid mini-brains created from induced pluripotent stem cells. |
| **2.7** | Are there other biologically plausible mechanisms of Zika infection leading congenital anomalies? | In vitro experiment (16): [23, 29, 37, 41, 42, 44-54] Sequencing and phylogenetics (3): [30, 55, 56] Biochemical/protein structure studies (3): [40, 57, 58] |  | 22 | 0 | Several authors demonstrate the effect of ZIKV in differentiating neuronal cells: [45] showed that undifferentiated neural cells lines were susceptible to ZIKV. [57] showed activation of caspase in different neural cell types, which plays a role in cell apoptosis. [47] compared gene expression by systematically assessing transcriptomes from ZIKV and DENV infected neural progenitor cells. Both the African and the Asian strain inhibited cell proliferation. [48] showed increased apoptosis and decreased proliferation in regions of expansion and folding of ZIKV infected cerebral organoids. [44, 49, 53] also demonstrate the infection of neuronal cells and the induced apoptosis. [56] identified in neuronal progenitor cells the expression of two new genes (MP0003861 and MP0002152) that seem to be related with nervous system/brain abnormalities. [57] show that cell viability was reduced due to ZIKV infection by induction of Caspase-3. [51] compared an Asian ZIKV strain with an African strain, where the African strain produced a higher infection rate and viral production in neural stem cells and astrocytes in vitro. [55] argue that ZIKV pathogenesis may be caused through precipitation of dysregulation in retinoic acid-dependent genes by introducing extra stretches of retinoic acid response elements consensus sequence repeats in the genome of developing brain cells. [30] showed using sequencing that in the more recent evolutionary history of ZIKV lineages, positive selection acted on NS5 and NS4B, this latter representing the preferential target where the N-terminal portion of NS4B might inhibit the immune response by inhibiting interferon response. [37] showed the susceptibility of ex vivo Hofbauer cells from human placentas to ZIKV infection. The authors argue that the migratory activities of Hofbauer cells may help in the dissemination of the virus into the foetal brain. [42] showed that ZIKV isolates in Brazil identifies more than 500 genes and proteins altered after viral infection in human neurospheres exposed to ZIKV. Prior to promote cell death, ZIKV impairs brain growth by arresting cell cycle and shutting down neurogenic programs during development of the central nervous system. [40] present an alternative hypothesis by demonstrating that in ZIKV positive brains, using mass spectrometry, bovine viral diarrhoea virus-like particles are present but not ZIKV particles. Arguing that other etiological agents might play a role. [50] demonstrate that co-infection with Herpes Simplex virus might enhance the ability of ZIKV to cross the placental barrier. [29] argued that haematogenous dissemination might be enhanced by the ability of ZIKV to infect endothelial cells. [58] shows that ZIKV, hCMV, and T. gondii are interconnected by common peptide sequences to human microcephaly-related proteins. [52] showed that ZIKV disrupts centriole biogenesis which is linked to a phenotype commonly associated with the autosomal recessive disorder, primary microcephaly (MCPH). [54] showed that the influence of ZIKV on the asymmetrical divisions of centrosomes in dividing human embryonic stem cells that might explain defects observed in ZIKV infection. |
| **Strength association** | | | | | | |
| **3.1** | How strong is the association between Zika infection and congenital anomalies at the individual level? | Case-control study (3): [59-61] Cohort study (2): [16, 18] | Brazil, United States, French Guiana | 5 | 0 | [59] reports on a case-control study in eight public hospitals in Recife, Brazil with 32 cases and 62 controls. 13 out of the 32 cases, and none of the 62 controls had laboratory-confirmed ZIKV infection; crude overall odds ratio (OR) was 55.5 (95% CI: 8.6-infinity). [60] reports on a case control study based on retrospective serology on Hawaii. ZIKV IgG antibody positive mothers had a higher likelihood of delivering babies with microcephaly than ZIKV negative mothers (OR 11.0 95% CI: 0.8-147.9, p = 0.083). Similarly, ZIKV IgM antibody positive mothers were also more likely to deliver babies with microcephaly than mothers who were negative for ZIKV IgM antibody (OR = 6.8, 95% CI = 0.2-195.1). [18] follows prospectively pregnant women and infants in Rio de Janeiro based on clinical manifestation and confirmation using PCR. 58 out of 117 women with PCR confirmed ZIKV had adverse congenital outcomes compared to 7 out of 58 women negative ZIKV PCR results. From this we calculate a risk ratio (RR) of 7.96 (95% CI 2.59-24.43). The RR for microcephaly (4 vs 0 cases) was 4.42 (95% CI 0.24-80.79). [16] describes a cohort in French Guiana. 27 out of 301 ZIKV positive women had adverse congenital outcomes versus 17 out of 399 in the unexposed group (RR 2.11 (95% CI 1.17-3.79)). Microcephaly occurred 5 times in the exposed group versus once in the unexposed group (RR 6.63 (95% CI 0.78-56.44)). |
| **3.2** | How strong is the association between Zika infection and congenital anomalies at the population level? | NO DATA | Brazil | 0 | 0 | NA |
| **Exclusion of alternatives** | | | | | | |
| **4.1** | Have other explanations/confounders of the association between Zika and congenital anomalies been excluded, such as TORCHS or other congenital infections? | Case report (11): [5-12, 22, 62, 63] Case series (5): [3, 24, 31-33] Cohort study (4): [14, 16-18] Case-control study (2): [59, 61] Ecological study/outbreak report (1): [19] | Spain, Brazil, Martinique, Suriname, Colombia, French Guiana, Slovenia, United States | 23 | 4 | TORCHS or other congenital infections have been assessed in 11 case reports [5-12, 22, 62, 63], 5 case series [3, 24, 31-33], 4 cohort studies [14, 16-18], one case-control study [59] and in one ecological study/outbreak report [19]. |
| **4.2** | Have other explanations/confounders of the association between Zika and congenital anomalies been excluded, such as maternal exposure to toxic chemicals (heavy metals, pesticides, drugs, alcohol, others)? | Case series (2): [31, 32] Cohort study (1): [14] Case report (2): [7, 10] | Brazil | 5 | 2 | Other explanations/confounders of the association between Zika and congenital anomalies such as maternal exposure to toxic chemicals (heavy metals, pesticides, drugs, alcohol, others) have been excluded in two case series [31, 32], two case reports [7, 10] and one cohort study [14]. |
| **4.3** | Have other explanations/confounders of the association between Zika and congenital anomalies been excluded, such as maternal or fetal malnutrition? | NO DATA |  | 0 | 0 | NA |
| **4.4** | Have other explanations/confounders of the association between Zika and congenital anomalies been excluded, such as hypoxic-ischaemic lesions? | NO DATA |  | 0 | 0 | NA |
| **4.5** | Have other explanations/confounders of the association between Zika and congenital anomalies been excluded, such as genetic conditions? | Case report (5): [5-7, 10, 12] Cohort study (1): [14] Case series (1): [32] | Martinique, Brazil, Suriname | 7 | 0 | Genetic conditions have been excluded in five case reports [5-7, 10, 12], one case series [32] and one cohort study [14]. |
| **4.6** | Have other explanations/confounders of the association between Zika and congenital anomalies been excluded, such as radiation? | NO DATA |  | 0 | 0 | NA |
| **5.1** | Does the intentional prevention/removal/elimination of Zika infection in individuals, e.g. by insect repellents, lead to a reduction in cases with congenital anomalies? | NO DATA |  | 0 | 0 | NA |
| **5.2** | Does the intentional removal/elimination/prevention of Zika at population-level, e.g. by vector control, lead to a reduction in cases with congenital anomalies? | NO DATA |  | 0 | 0 | NA |
| **5.3** | Does a natural removal/elimination/prevention of Zika at population-level, e.g. increase in immune individuals or decrease in vector abundance lead to a reduction in cases with congenital anomalies? | NO DATA |  | 0 | 0 | NA |
| **Dose response** | | | | | | |
| **6.1** | Are the risk and the clinical severity of congenital anomalies associated with the viral load in maternal serum, urine, the placenta and/or amniotic fluid? | NO DATA |  | 0 | 0 | NA |
| **6.2** | Are the risk and the clinical severity of congenital anomalies associated with the clinical severity (including being asymptomatic) of Zika infection in the mother? | NO DATA |  | 0 | 1 | [35] compares symptomatic with asymptomatic pregnant women in a cohort of 442 women from the United States. No significant difference in adverse congenital outcome is noted between these groups. Birth defects were reported in 16 out of 271 (6% 95% CI:4-9%) pregnant asymptomatic women and 10 out of 167 (6% 95% CI: 3-11%) symptomatic pregnant women. |
| **Animal experiments** | | | | | | |
| **7.1** | Does inoculation of pregnant female animals with Zika virus cause congenital anomalies in the offspring? | Animal experiment (3): [64-66] |  | 3 | 0 | Pregnant female animals infected with ZIKV were demonstrated to have affected offspring after infection with ZIKV in mice and non-human primates. [64, 65] shows that intravenous or intravaginal infection of pregnant mice with ZIKV leads to foetal growth restriction and brain involvement of the foetus. [66] shows that inoculation of pregnant Pigtail Macaques leads to foetal brain injuries. |
| **7.2** | Does intracerebral inoculation of newborn animals with Zika virus lead to Zika virus virus replication in the CNS? | Animal experiment (3): [67-69] | French Polynesia | 3 | 0 | [68, 69] show that after intracranial inoculation of mice ZIKV successfully replicates; rapid brain growth seems particularly susceptible to ZIKV infection. Additionally, [68] shows a damages blood-brain barrier after ZIKV infection. [67] shows that ex vivo foetal brain tissue is susceptible to ZIKV infection with a tropism for neural stem cells. |
| **7.3** | Does any other route of inoculation of newborn animals with Zika virus lead to Zika virus virus replication in the CNS? | Animal experiment (3): [65, 70, 71] |  | 3 | 0 |  |
| **7.4** | Do other experiments with animals or animal-derived cells support the association of Zika infection and congenital anomalies? | In vitro experiment (2): [46, 50] Animal experiment (6): [66, 68-72] |  | 8 | 0 | Two in vitro experiments and seven animal studies were included. In vitro herpes simplex virus enhances the sensitivity of trophoblasts to ZIKV [50]. [46] shows that from the tested animal cell lines mostly kidney cells were susceptible to ZIKV. However, few cells of cells of neural origin were tested. [66] shows that inoculation of non-human primates leads to foetal brain injuries. [70] shows that 10-day old Ifnar knockout mice display several neurological symptoms after ZIKV inoculation, they showed bilateral paralysis and succumbed to disease. 1-day-old wild type mice develop neurological symptoms less severe than the Ifnar knockout mice. [68] demonstrates, using gene analyses, that dysregulation of genes associated with immune responses occurs in ZIKV infected brains of mice. In early post-natal mice after intracerebral inoculation with ZIKV a widespread distribution of virus is noted [69]. AXL is shown to be distributed throughout the brain. The authors conclude that periods of high growth rate may be particularly vulnerable to neurological effects of ZIKV infection [69]. [72] shows that diverse isolates of ZIKV, were able to replicate in mouse embryonic brain slice preparations, with differences in viral pathology. The authors observed a severe decrease in mitotic events and evidence for subsequent defects in post mitotic neuronal migration and viability as well. [71] Chick embryos were infected with ZIKV and longitudinally monitored by magnetic resonance imaging. Enlarged ventricles and stunted cortical growth was observed. |
| **Analogy** | | | | | | |
| **8.1** | Do other flaviviruses or arboviruses cause congenital anomalies and by which mechanism(s)? | Case series (1): [73] |  | 1 | 0 | CHIKV appeared to be vertically transmissible and lead to adverse congenital outcomes in a prospective study in the Americas [73]. |
| **8.2** | Do other pathogens cause congenital anomalies and by which mechanism(s)? | Ecological study/outbreak report (1): [74] |  | 1 | 0 | [74] provides a review of the TORCH infections, the possible explanations in shift in ZIKV and a change in tropism. This is compared with DENV and WNV. |
| **8.3** | Which pathogen or host factors facilitate the development of congenital anomalies? | Ecological study/outbreak report (1): [74] |  | 1 | 0 | [74] provides a review of the TORCH infections, the possible explanations in shift in ZIKV and a change in tropism. This is compared with DENV and WNV. |
| **Specificity** | | | | | | |
| **9.1** | Are there pathological findings in cases with congenital anomalies that are specific for Zika infection? | Review (1): [75] |  | 4 | 0 | [75] reviews published reports up to 30.09.2016 and conclude that five aspects of adverse congenital outcomes are either unique to ZIKV or rarely seen in other infections. These features are: severe microcephaly with overlapping cranial structures, subcortical location of brain calcifications, macular scarring and retinal mottling, congenital contractures and early pyramidal and extrapyramidal symptoms. The authors therefore conclude that a distinct congenital Zika syndrome phenotype can be distinguished. |
| **Consistency** | | | | | | |
| **10.1** | Is the association between Zika cases and cases with congenital anomalies disorders consistently found across different geographical regions? | Case series (1): [76] Ecological study/outbreak report (2): [77, 78] | Brazil | 3 | 1 | [77] and [78] report that 29 countries have reported microcephaly and other CNS malformations that are potentially associated with ZIKV infection. Although the proportion of microcephaly cases compared to either cumulative confirmed cases of ZIKV or cumulative suspected cases of ZIKV varies over different regions (<http://www.paho.org/hq/index.php?option=com_docman&task=doc_view&Itemid=270&gid=37618&lang=en>). |
| **10.2** | Is the association between Zika cases and cases with congenital anomalies consistently found across different populations/subpopulations? | NO DATA |  | 0 | 0 |  |
| **10.3** | Is the association between Zika cases and cases with congenital anomalies consistently found across different Zika lineages/strains? | NO DATA |  | 0 | 1 |  |
| **10.4** | Is the association between Zika cases and cases with congenital anomalies consistently found across different study designs? | NO DATA |  | 0 | 0 | NA |

# References

1. Meaney-Delman D, Oduyebo T, Polen KN, White JL, Bingham AM, Slavinski SA, et al. Prolonged Detection of Zika Virus RNA in Pregnant Women. Obstet Gynecol. 2016;128(4):724-30. doi: 10.1097/AOG.0000000000001625. PubMed PMID: 27479770.

2. Carvalho FH, Cordeiro KM, Peixoto AB, Tonni G, Moron AF, Feitosa FE, et al. Associated ultrasonographic findings in fetuses with microcephaly because of suspected Zika virus (ZIKV) infection during pregnancy. Prenat Diagn. 2016;36(9):882-7. doi: 10.1002/pd.4882. PubMed PMID: 27491635.

3. van der Linden V, Filho EL, Lins OG, van der Linden A, Aragao Mde F, Brainer-Lima AM, et al. Congenital Zika syndrome with arthrogryposis: retrospective case series study. BMJ. 2016;354(8):i3899. doi: 10.1136/bmj.i3899. PubMed PMID: 27509902; PubMed Central PMCID: PMCPMC4979356.

4. Vargas A, Saad E, Dimech GS, Santos RH, Sivini MA, Albuquerque LC, et al. Characteristics of the first cases of microcephaly possibly related to Zika virus reported in the Metropolitan Region of Recife, Pernambuco State, Brazil. Epidemiol Serv Saude. 2016;25(4):691-700. doi: 10.5123/S1679-49742016000400003. PubMed PMID: 27869982.

5. Schaub B, Monthieux A, Najioullah F, Adenet C, Muller F, Cesaire R. Persistent maternal Zika viremia: a marker of fetal infection. Ultrasound Obstet Gynecol. 2017;49(5):658-60. doi: 10.1002/uog.17210. PubMed PMID: 27485955.

6. van der Eijk AA, van Genderen PJ, Verdijk RM, Reusken CB, Mogling R, van Kampen JJ, et al. Miscarriage Associated with Zika Virus Infection. N Engl J Med. 2016;375(10):1002-4. doi: 10.1056/NEJMc1605898. PubMed PMID: 27463941.

7. Leal MC, Muniz LF, Caldas Neto SD, van der Linden V, Ramos RC. Sensorineural hearing loss in a case of congenital Zika virus. Braz J Otorhinolaryngol. 2016;0:0. doi: 10.1016/j.bjorl.2016.06.001. PubMed PMID: 27444419.

8. Suy A, Sulleiro E, Rodo C, Vazquez E, Bocanegra C, Molina I, et al. Prolonged Zika Virus Viremia during Pregnancy. N Engl J Med. 2016;375(26):2611-3. doi: 10.1056/NEJMc1607580. PubMed PMID: 27959695.

9. de Paula Freitas B, Ko AI, Khouri R, Mayoral M, Henriques DF, Maia M, et al. Glaucoma and Congenital Zika Syndrome. Ophthalmology. 2017;124(3):407-8. doi: 10.1016/j.ophtha.2016.10.004. PubMed PMID: 27914834.

10. Campos AG, Lira RP, Arantes TE. Optical coherence tomography of macular atrophy associated with microcephaly and presumed intrauterine Zika virus infection. Arq Bras Oftalmol. 2016;79(6):400-1. doi: 10.5935/0004-2749.20160112. PubMed PMID: 28076569.

11. Ventura CV, Fernandez MP, Gonzalez IA, Rivera-Hernandez DM, Lopez-Alberola R, Peinado M, et al. First Travel-Associated Congenital Zika Syndrome in the US: Ocular and Neurological Findings in the Absence of Microcephaly. Ophthalmic Surg Lasers Imaging Retina. 2016;47(10):952-5. doi: 10.3928/23258160-20161004-09. PubMed PMID: 27759862.

12. Werner H, Fazecas T, Guedes B, Lopes Dos Santos J, Daltro P, Tonni G, et al. Intrauterine Zika virus infection and microcephaly: correlation of perinatal imaging and three-dimensional virtual physical models. Ultrasound Obstet Gynecol. 2016;47(5):657-60. doi: 10.1002/uog.15901. PubMed PMID: 26923098.

13. Bhadelia N. Prospective cohort study of pregnant Brazilian women elucidates link between Zika virus infection and fetal abnormalities. Evid Based Med. 2016;21(5):193. doi: 10.1136/ebmed-2016-110476. PubMed PMID: 27495823.

14. Leal MC, Muniz LF, Ferreira TS, Santos CM, Almeida LC, Van Der Linden V, et al. Hearing Loss in Infants with Microcephaly and Evidence of Congenital Zika Virus Infection - Brazil, November 2015-May 2016. MMWR Morb Mortal Wkly Rep. 2016;65(34):917-9. doi: 10.15585/mmwr.mm6534e3. PubMed PMID: 27585248.

15. Sanin-Blair JCA, Gutierrez-Marin J, Campo-Campo M, Garcia-Posada R, Londo. Fetal ultrasound findings in zika infection. Preliminary report in a Colombian selected population. Journal of Maternal-Fetal and Neonatal Medicine. 2016;29(2):163-4.

16. Pomar L, Malinger G, Benoist G, Carles G, Ville Y, Rousset D, et al. Association between Zika virus and fetopathy: a prospective cohort study in French Guiana. Ultrasound Obstet Gynecol. 2017;49(6):729-36. doi: 10.1002/uog.17404. PubMed PMID: 28078779.

17. Moura da Silva AA, Ganz JS, Sousa PD, Doriqui MJ, Ribeiro MR, Branco MD, et al. Early Growth and Neurologic Outcomes of Infants with Probable Congenital Zika Virus Syndrome. Emerg Infect Dis. 2016;22(11):1953-6. doi: 10.3201/eid2211.160956. PubMed PMID: 27767931; PubMed Central PMCID: PMCPMC5088045.

18. Brasil P, Pereira JP, Jr., Moreira ME, Ribeiro Nogueira RM, Damasceno L, Wakimoto M, et al. Zika Virus Infection in Pregnant Women in Rio de Janeiro. N Engl J Med. 2016;375(24):2321-34. doi: 10.1056/NEJMoa1602412. PubMed PMID: 26943629; PubMed Central PMCID: PMCPMC5323261.

19. Cuevas EL, Tong VT, Rozo N, Valencia D, Pacheco O, Gilboa SM, et al. Preliminary Report of Microcephaly Potentially Associated with Zika Virus Infection During Pregnancy - Colombia, January-November 2016. MMWR Morb Mortal Wkly Rep. 2016;65(49):1409-13. doi: 10.15585/mmwr.mm6549e1. PubMed PMID: 27977645.

20. Herrera K, Bernasko J, Garry D, Vahanian S, Kaplan C. Vertical transmission of Zika virus (ZIKV) in early pregnancy: two cases, two different courses. Case Rep Perinat Med. 2016;5(2):131-3. doi: 10.1515/crpm-2016-0027.

21. Magalhaes-Barbosa MC, Prata-Barbosa A, Robaina JR, Raymundo CE, Lima-Setta F, Cunha AJ. Trends of the microcephaly and Zika virus outbreak in Brazil, January-July 2016. Travel Med Infect Dis. 2016;14(5):458-63. doi: 10.1016/j.tmaid.2016.09.006. PubMed PMID: 27702683.

22. Perez S, Tato R, Cabrera JJ, Lopez A, Robles O, Paz E, et al. Confirmed case of Zika virus congenital infection, Spain, March 2016. Euro Surveill. 2016;21(24):0. doi: 10.2807/1560-7917.ES.2016.21.24.30261. PubMed PMID: 27336620.

23. Weisblum Y, Oiknine-Djian E, Vorontsov OM, Haimov-Kochman R, Zakay-Rones Z, Meir K, et al. Zika Virus Infects Early- and Midgestation Human Maternal Decidual Tissues, Inducing Distinct Innate Tissue Responses in the Maternal-Fetal Interface. J Virol. 2017;91(4):pii: e01905-16. doi: 10.1128/JVI.01905-16. PubMed PMID: 27974560; PubMed Central PMCID: PMCPMC5286880.

24. Soares de Souza A, Moraes Dias C, Braga FD, Terzian AC, Estofolete CF, Oliani AH, et al. Fetal Infection by Zika Virus in the Third Trimester: Report of 2 Cases. Clin Infect Dis. 2016;63(12):1622-5. doi: 10.1093/cid/ciw613. PubMed PMID: 27601223.

25. Tabata T, Petitt M, Puerta-Guardo H, Michlmayr D, Wang C, Fang-Hoover J, et al. Zika Virus Targets Different Primary Human Placental Cells, Suggesting Two Routes for Vertical Transmission. Cell Host Microbe. 2016;20(2):155-66. doi: 10.1016/j.chom.2016.07.002. PubMed PMID: 27443522; PubMed Central PMCID: PMCPMC5257282.

26. Onorati M, Li Z, Liu F, Sousa AMM, Nakagawa N, Li M, et al. Zika Virus Disrupts Phospho-TBK1 Localization and Mitosis in Human Neuroepithelial Stem Cells and Radial Glia. Cell Rep. 2016;16(10):2576-92. doi: 10.1016/j.celrep.2016.08.038. PubMed PMID: 27568284; PubMed Central PMCID: PMCPMC5135012.

27. Retallack H, Di Lullo E, Arias C, Knopp KA, Sandoval-Espinosa C, Laurie MT, et al. Zika Virus in the Human Placenta and Developing Brain: Cell Tropism and Drug Inhibition. bioRxiv. 2016;0:058883. doi: 10.1101/058883.

28. Retallack H, Di Lullo E, Arias C, Knopp KA, Laurie MT, Sandoval-Espinosa C, et al. Zika virus cell tropism in the developing human brain and inhibition by azithromycin. Proc Natl Acad Sci U S A. 2016;113(50):14408-13. doi: 10.1073/pnas.1618029113. PubMed PMID: 27911847; PubMed Central PMCID: PMCPMC5167169.

29. Liu S, DeLalio LJ, Isakson BE, Wang TT. AXL-Mediated Productive Infection of Human Endothelial Cells by Zika Virus. Circ Res. 2016;119(11):1183-9. doi: 10.1161/CIRCRESAHA.116.309866. PubMed PMID: 27650556; PubMed Central PMCID: PMCPMC5215127.

30. Sironi M, Forni D, Clerici M, Cagliani R. Nonstructural Proteins Are Preferential Positive Selection Targets in Zika Virus and Related Flaviviruses. PLoS Negl Trop Dis. 2016;10(9):e0004978. doi: 10.1371/journal.pntd.0004978. PubMed PMID: 27588756; PubMed Central PMCID: PMCPMC5010288.

31. Martines RB, Bhatnagar J, de Oliveira Ramos AM, Davi HP, Iglezias SD, Kanamura CT, et al. Pathology of congenital Zika syndrome in Brazil: a case series. Lancet. 2016;388(10047):898-904. doi: 10.1016/S0140-6736(16)30883-2. PubMed PMID: 27372395.

32. Melo AS, Aguiar RS, Amorim MM, Arruda MB, Melo FO, Ribeiro ST, et al. Congenital Zika Virus Infection: Beyond Neonatal Microcephaly. JAMA Neurol. 2016;73(12):1407-16. doi: 10.1001/jamaneurol.2016.3720. PubMed PMID: 27695855.

33. van der Linden V, Pessoa A, Dobyns W, Barkovich AJ, Junior HV, Filho EL, et al. Description of 13 Infants Born During October 2015-January 2016 With Congenital Zika Virus Infection Without Microcephaly at Birth - Brazil. MMWR Morb Mortal Wkly Rep. 2016;65(47):1343-8. doi: 10.15585/mmwr.mm6547e2. PubMed PMID: 27906905.

34. Bhatnagar J, Rabeneck DB, Martines RB, Reagan-Steiner S, Ermias Y, Estetter LB, et al. Zika Virus RNA Replication and Persistence in Brain and Placental Tissue. Emerg Infect Dis. 2017;23(3):405-14. doi: 10.3201/eid2303.161499. PubMed PMID: 27959260; PubMed Central PMCID: PMCPMC5382738.

35. Honein MA, Dawson AL, Petersen EE, Jones AM, Lee EH, Yazdy MM, et al. Birth Defects Among Fetuses and Infants of US Women With Evidence of Possible Zika Virus Infection During Pregnancy. JAMA. 2017;317(1):59-68. doi: 10.1001/jama.2016.19006. PubMed PMID: 27960197.

36. Tsetsarkin KA, Kenney H, Chen R, Liu G, Manukyan H, Whitehead SS, et al. A Full-Length Infectious cDNA Clone of Zika Virus from the 2015 Epidemic in Brazil as a Genetic Platform for Studies of Virus-Host Interactions and Vaccine Development. MBio. 2016;7(4):e01114-16. doi: 10.1128/mBio.01114-16. PubMed PMID: 27555311; PubMed Central PMCID: PMCPMC4999549.

37. Jurado KA, Simoni MK, Tang Z, Uraki R, Hwang J, Householder S, et al. Zika virus productively infects primary human placenta-specific macrophages. JCI Insight. 2016;1(13):0. doi: 10.1172/jci.insight.88461. PubMed PMID: 27595140; PubMed Central PMCID: PMCPMC5007065.

38. El Costa H, Gouilly J, Mansuy JM, Chen Q, Levy C, Cartron G, et al. ZIKA virus reveals broad tissue and cell tropism during the first trimester of pregnancy. Sci Rep. 2016;6:35296. doi: 10.1038/srep35296. PubMed PMID: 27759009; PubMed Central PMCID: PMCPMC5069472.

39. Hanners NW, Eitson JL, Usui N, Richardson RB, Wexler EM, Konopka G, et al. Western Zika Virus in Human Fetal Neural Progenitors Persists Long Term with Partial Cytopathic and Limited Immunogenic Effects. Cell Rep. 2016;15(11):2315-22. doi: 10.1016/j.celrep.2016.05.075. PubMed PMID: 27268504.

40. Nogueira FCS, Velasquez E, Melo ASO, Domont GB. Zika virus may not be alone: proteomics associates a bovine-like viral diarrhea virus to microcephaly. bioRxiv. 2016;0:062596. doi: 10.1101/062596. PubMed PMID: 0.

41. Liang Q, Luo Z, Zeng J, Chen W, Foo SS, Lee SA, et al. Zika Virus NS4A and NS4B Proteins Deregulate Akt-mTOR Signaling in Human Fetal Neural Stem Cells to Inhibit Neurogenesis and Induce Autophagy. Cell Stem Cell. 2016;19(5):663-71. doi: 10.1016/j.stem.2016.07.019. PubMed PMID: 27524440; PubMed Central PMCID: PMCPMC5144538.

42. Garcez PP, Nascimento JM, Mota de Vasconcelos J, Madeiro da Costa R, Delvecchio R, Trindade P, et al. Combined proteome and transcriptome analyses reveal that Zika virus circulating in Brazil alters cell cycle and neurogenic programmes in human neurospheres. PeerJ Preprints. 2016;0:0. doi: 10.7287/peerj.preprints.2033v1. PubMed PMID: 0.

43. Bayless NL, Greenberg RS, Swigut T, Wysocka J, Blish CA. Zika Virus Infection Induces Cranial Neural Crest Cells to Produce Cytokines at Levels Detrimental for Neurogenesis. Cell Host Microbe. 2016;20(4):423-8. doi: 10.1016/j.chom.2016.09.006. PubMed PMID: 27693308; PubMed Central PMCID: PMCPMC5113290.

44. Barreras P, Pamies D, Kumar A, Munoz LS, Parra B, Abreu C, et al. 2016 Annual Meetings. Ann Neurol. 2016;80(s20):S1-S432. doi: 10.1002/ana.24759. PubMed PMID: 27739587.

45. Hughes BW, Addanki KC, Sriskanda AN, McLean E, Bagasra O. Infectivity of Immature Neurons to Zika Virus: A Link to Congenital Zika Syndrome. EBioMedicine. 2016;10(10038):65-70. doi: 10.1016/j.ebiom.2016.06.026. PubMed PMID: 27364784; PubMed Central PMCID: PMCPMC5006602.

46. Chan JF, Yip CC, Tsang JO, Tee KM, Cai JP, Chik KK, et al. Differential cell line susceptibility to the emerging Zika virus: implications for disease pathogenesis, non-vector-borne human transmission and animal reservoirs. Emerg Microbes Infect. 2016;5:e93. doi: 10.1038/emi.2016.99. PubMed PMID: 27553173; PubMed Central PMCID: PMCPMC5034105.

47. Zhang F, Hammack C, Ogden SC, Cheng Y, Lee EM, Wen Z, et al. Molecular signatures associated with ZIKV exposure in human cortical neural progenitors. Nucleic Acids Res. 2016;44(18):8610-20. doi: 10.1093/nar/gkw765. PubMed PMID: 27580721; PubMed Central PMCID: PMCPMC5063002.

48. Li Y, Muffat J, Omer A, Bosch I, Lancaster MA, Sur M, et al. Induction of Expansion and Folding in Human Cerebral Organoids. Cell Stem Cell. 2017;20(3):385-96 e3. doi: 10.1016/j.stem.2016.11.017. PubMed PMID: 28041895.

49. Souza BS, Sampaio GL, Pereira CS, Campos GS, Sardi SI, Freitas LA, et al. Zika virus infection induces mitosis abnormalities and apoptotic cell death of human neural progenitor cells. Sci Rep. 2016;6:39775. doi: 10.1038/srep39775. PubMed PMID: 28008958; PubMed Central PMCID: PMCPMC5180086.

50. Aldo P, You Y, Szigeti K, Horvath TL, Lindenbach B, Mor G. HSV-2 enhances ZIKV infection of the placenta and induces apoptosis in first-trimester trophoblast cells. Am J Reprod Immunol. 2016;76(5):348-57. doi: 10.1111/aji.12578. PubMed PMID: 27613665.

51. Simonin Y, Loustalot F, Desmetz C, Foulongne V, Constant O, Fournier-Wirth C, et al. Zika Virus Strains Potentially Display Different Infectious Profiles in Human Neural Cells. EBioMedicine. 2016;12:161-9. doi: 10.1016/j.ebiom.2016.09.020. PubMed PMID: 27688094; PubMed Central PMCID: PMCPMC5078617.

52. Kodani AT, Reiter JF, Knopp K, DeRisi J. Zika infection disrupts centriole biogenesis. Mol Biol Cell. 2016;27(25):4182-3. doi: 10.1091/mbc.E16-10-0736. PubMed PMID: WOS:000394259500375.

53. Veilleux C, Eugenin E. Characterization of Zika virus infection in Human Astrocytes. Journal of NeuroVirology. 2016;22:S78-S9. doi: 10.1007/s13365-016-0478-8.

54. Yaffe Y, Dellibovi-Ragheb TA, Chen Y, Elkabetz Y, Altan-Bonnet N. Board Number: B456 The role of centrosomes in the pathogenesis of Zika virus (ZIKV)-related microcephaly. Mol Biol Cell. 2016;27(25):4919. doi: 10.1091/mbc.E16-10-0736. PubMed PMID: NA.

55. Kumar A, Singh HN, Pareek V, Raza K, Dantham S, Kumar P, et al. A Possible Mechanism of Zika Virus Associated Microcephaly: Imperative Role of Retinoic Acid Response Element (RARE) Consensus Sequence Repeats in the Viral Genome. Front Hum Neurosci. 2016;10:403. doi: 10.3389/fnhum.2016.00403. PubMed PMID: 27555815; PubMed Central PMCID: PMCPMC4977292.

56. Wang Z, Ma'ayan A. An open RNA-Seq data analysis pipeline tutorial with an example of reprocessing data from a recent Zika virus study. F1000Res. 2016;5(1574):1574. doi: 10.12688/f1000research.9110.1. PubMed PMID: 27583132; PubMed Central PMCID: PMCPMC4972086.

57. Xu M, Lee EM, Wen Z, Cheng Y, Huang WK, Qian X, et al. Identification of small-molecule inhibitors of Zika virus infection and induced neural cell death via a drug repurposing screen. Nat Med. 2016;22(10):1101-7. doi: 10.1038/nm.4184. PubMed PMID: 27571349; PubMed Central PMCID: PMCPMC5386783.

58. Lucchese G, Kanduc D. Minimal immune determinants connect Zika virus, human Cytomegalovirus, and Toxoplasma gondii to microcephaly-related human proteins. Am J Reprod Immunol. 2017;77(2). doi: 10.1111/aji.12608. PubMed PMID: 27878897.

59. Thalia Velho Barreto A, Laura CR, Ricardo AX, Demócrito BM-F, Ulisses RM, Ana Paula LM, et al. Microcephaly and Zika infection: Preliminary Report of a Case-Control Study. Lancet. 2016;16(12):1356-63. doi: 10.1016/S1473-3099(16)30318-8. PubMed PMID: 27641777.

60. Kumar M, Ching L, Astern J, Lim E, Stokes AJ, Melish M, et al. Prevalence of Antibodies to Zika Virus in Mothers from Hawaii Who Delivered Babies with and without Microcephaly between 2009-2012. PLoS Negl Trop Dis. 2016;10(12):e0005262. doi: 10.1371/journal.pntd.0005262. PubMed PMID: 27997547; PubMed Central PMCID: PMCPMC5215948.

61. de Araujo TVB, Rodrigues LC, de Alencar Ximenes RA, de Barros Miranda-Filho D, Montarroyos UR, de Melo APL, et al. Association between Zika virus infection and microcephaly in Brazil, January to May, 2016: preliminary report of a case-control study. Lancet Infect Dis. 2016;16(12):1356-63. doi: 10.1016/S1473-3099(16)30318-8. PubMed PMID: 27641777.

62. Oliveira DB, Almeida FJ, Durigon EL, Mendes EA, Braconi CT, Marchetti I, et al. Prolonged Shedding of Zika Virus Associated with Congenital Infection. N Engl J Med. 2016;375(12):1202-4. doi: 10.1056/NEJMc1607583. PubMed PMID: 27653589.

63. Joao EC, da Silveira Gouvea MI, de Lourdes Benamor Teixeira M, Mendes-Silva W, Esteves JS, Santos EM, et al. Zika Virus Infection Associated with Congenital Birth Defects in a HIV-Infected Pregnant Woman. Pediatr Infect Dis J. 2016;36(5):500-1. doi: 10.1097/INF.0000000000001482. PubMed PMID: 27997515.

64. Cugola FR, Fernandes IR, Russo FB, Freitas BC, Dias JL, Guimaraes KP, et al. The Brazilian Zika virus strain causes birth defects in experimental models. Nature. 2016;534(7606):267-71. doi: 10.1038/nature18296. PubMed PMID: 27279226.

65. Yockey LJ, Varela L, Rakib T, Khoury-Hanold W, Fink SL, Stutz B, et al. Vaginal Exposure to Zika Virus during Pregnancy Leads to Fetal Brain Infection. Cell. 2016;166(5):1247-56 e4. doi: 10.1016/j.cell.2016.08.004. PubMed PMID: 27565347; PubMed Central PMCID: PMCPMC5006689.

66. Adams Waldorf KM, Stencel-Baerenwald JE, Kapur RP, Studholme C, Boldenow E, Vornhagen J, et al. Fetal brain lesions after subcutaneous inoculation of Zika virus in a pregnant nonhuman primate. Nat Med. 2016;22(11):1256-9. doi: 10.1038/nm.4193. PubMed PMID: 27618651; PubMed Central PMCID: PMCPMC5365281.

67. Brault JB, Khou C, Basset J, Coquand L, Fraisier V, Frenkiel MP, et al. Comparative Analysis Between Flaviviruses Reveals Specific Neural Stem Cell Tropism for Zika Virus in the Mouse Developing Neocortex. EBioMedicine. 2016;10(2):71-6. doi: 10.1016/j.ebiom.2016.07.018. PubMed PMID: 27453325; PubMed Central PMCID: PMCPMC5006693.

68. Shao Q, Herrlinger S, Yang SL, Lai F, Moore JM, Brindley MA, et al. Zika virus infection disrupts neurovascular development and results in postnatal microcephaly with brain damage. Development. 2016;143(22):4127-36. doi: 10.1242/dev.143768. PubMed PMID: 27729407; PubMed Central PMCID: PMCPMC5117220.

69. Huang WC, Abraham R, Shim BS, Choe H, Page DT. Zika virus infection during the period of maximal brain growth causes microcephaly and corticospinal neuron apoptosis in wild type mice. Sci Rep. 2016;6:34793. doi: 10.1038/srep34793. PubMed PMID: 27713505; PubMed Central PMCID: PMCPMC5054421.

70. Manangeeswaran M, Ireland DD, Verthelyi D. Zika (PRVABC59) Infection Is Associated with T cell Infiltration and Neurodegeneration in CNS of Immunocompetent Neonatal C57Bl/6 Mice. PLoS Pathog. 2016;12(11):e1006004. doi: 10.1371/journal.ppat.1006004. PubMed PMID: 27855206; PubMed Central PMCID: PMCPMC5113993.

71. Goodfellow FT, Tesla B, Simchick G, Zhao Q, Hodge T, Brindley MA, et al. Zika Virus Induced Mortality and Microcephaly in Chicken Embryos. Stem Cells Dev. 2016;25(22):1691-7. doi: 10.1089/scd.2016.0231. PubMed PMID: 27627457.

72. Doobin DJ, Rosenfeld A, Carabalona A, Racaniello V, Vallee RB. Genetic and infectious causes of microcephaly caused by NDE1 mutations and Zika virus. Mol Biol Cell. 2016;27(25):4129-30. doi: 10.1091/mbc.E16-10-0736. PubMed PMID: WOS:000394259500291.

73. Torres JR, Falleiros-Arlant LH, Duenas L, Pleitez-Navarrete J, Salgado DM, Castillo JB. Congenital and perinatal complications of chikungunya fever: a Latin American experience. Int J Infect Dis. 2016;51:85-8. Epub 2016/10/26. doi: 10.1016/j.ijid.2016.09.009. PubMed PMID: 27619845.

74. Panchaud A, Stojanov M, Ammerdorffer A, Vouga M, Baud D. Emerging Role of Zika Virus in Adverse Fetal and Neonatal Outcomes. Clin Microbiol Rev. 2016;29(3):659-94. doi: 10.1128/cmr.00014-16. PubMed PMID: 27281741.

75. Moore CA, Staples JE, Dobyns WB, Pessoa A, Ventura CV, Fonseca EB, et al. Characterizing the Pattern of Anomalies in Congenital Zika Syndrome for Pediatric Clinicians. JAMA Pediatr. 2017;171(3):288-95. doi: 10.1001/jamapediatrics.2016.3982. PubMed PMID: 27812690; PubMed Central PMCID: PMCPMC5561417.

76. Franca GV, Schuler-Faccini L, Oliveira WK, Henriques CM, Carmo EH, Pedi VD, et al. Congenital Zika virus syndrome in Brazil: a case series of the first 1501 livebirths with complete investigation. Lancet. 2016;388(10047):891-7. doi: 10.1016/S0140-6736(16)30902-3. PubMed PMID: 27372398.

77. World Health Organization. WHO | Zika situation report 05-01-2017 2017 [18/12/2017]. Available from: <http://www.who.int/emergencies/zika-virus/situation-report/05-january-2017/en/>.

78. Pan American Health Organization P. Epidemiological Update. Zika virus infection - 12 January 2017 2017 [20/11/2017]. Available from: <http://www.paho.org/hq/index.php?option=com_docman&task=doc_download&gid=37671&lang=en>.
