# Supplementary material for: Zika virus infection as a cause of congenital brain abnormalities and Guillain-Barré syndrome: From systematic review to living systematic review
Source: F1000Res. 2018 Feb 15;7:196. [Version 1] doi: 10.12688/f1000research.13704.1 (PMC6290976; doi:10.12688/f1000research.13704.1)
Supplement: Supplementary file 3 [file f1000research-7-14886-s0002.tgz › a81d0c18-7c01-40aa-b05c-36338a3419c7.docx]

# Supplementary Table 3 – Evidence table GBS/ITP update 1

| **Question No** | **Causality dimension + question** | **Study type (number) references** | **Country of study** | **Support of causality dimension** | **Evidence against causality** | **Summary** |
| --- | --- | --- | --- | --- | --- | --- |
| **Temporality** | | | | | | |
| **1.1a** | Does Zika infection precede the development of autoimmune-mediated disorders at individual level? | Case series (9): [1-9] Ecological study/outbreak report (2): [10, 11] Case report (5): [12-16] Case-control study (1): [17] | Ecuador, France, Puerto Rico, French Guiana, Venezuela, Suriname, Guadeloupe, Colombia, El Salvador, Guyana, United States, Brazil | 17 | 0 | In nine case series [1-9], two ecological studies or outbreak reports [10, 11], five case reports [12-16] and one case-control study [17] ZIKV infection preceded the development of autoimmune-mediated disorders. Three studies reported on ITP [3, 5, 14], the rest of the studies on GBS [1, 2, 4, 6-13, 15-17]. [18] show that the peak of GBS cases precedes the peak of ZIKV infections by two weeks when surveillance data from seven different countries (Brazil, Colombia, Dominican Republic, El Salvador, Honduras, Suriname and Venezuela) are combined. This discrepancy might be due difference in reporting between ZIKV incidence and GBS incidence. However, this finding contradicts that ZIKV infection precedes GBS. |
| **1.1b** | Is there a consistent time-dependent relationship between the occurrence of Zika cases and cases with GBS/ITP at population-level? | Ecological study/outbreak report (2): [18, 19] Cross-sectional study (1): [20] Case series (1): [8] | Colombia, Brazil, Dominican Republic, El Salvador, Honduras, Suriname, Venezuela | 4 | 0 | Larger studies (case series and a case control study with n=34, n=19 and n=41, n=29 likely and confirmed ZIKV cases with GBS) reported median duration between onset of symptoms of ZIKV and onset of GBS between 5 and 10 days [2, 7, 8, 17]. Smaller case series and case reports agreed with these results and the duration between onset of ZIKV symptoms and onset of adverse autoimmune outcomes did not exceed 17 days [2]. [8] distinguishes between a para-infectious (20/42) onset of GBS and a post-infectious onset of GBS (22/42), where ZIKV was followed by an asymptomatic period after which neurological symptoms appeared. |
| **1.2** | Is the interval between exposure to Zika and occurrence of symptoms typical for para- or post-infectious autoimmune-mediated disorders? | Case series (8): [1, 2, 4-9] Case report (6): [12-16, 21] Case-control study (1): [17] | Ecuador, Puerto Rico, Venezuela, Suriname, Guadeloupe, Colombia, El Salvador, Guyana, New Zealand, Brazil, United States | 15 | 0 | ITP: [5] 5 days (7 cases), [14] 2 days (1 case). GBS: for 107 cases of likely or confirmed GBS cases with likely or confirmed ZIKV described in 11 studies, information on the duration between onset of ZIKV symptoms and onset of GBS was available [2, 4, 6-9, 12, 13, 15, 16, 21]. The intervals reported between exposure to Zika and occurrence of symptoms are typical for para- or post-infectious autoimmune-mediated disorders. |
| **Biological plausibility** | | | | | | |
| **2.1** | Do Zika virus epitopes mimic host antigens (molecular mimicry)? | NO DATA |  | 0 | 0 | NA |
| **2.2** | Does Zika infection lead to an increased in detectable autoreactive immune cells or autoreactive antibodies? | NO DATA |  | 0 | 0 | NA |
| **2.3** | Are there other biologically plausible mechanisms of Zika infection leading to GBS/ITP? | NO DATA |  | 0 | 0 | NA |
| **Strength of association** | | | | | | |
| **3.1** | How strong is the association between Zika infection and GBS/ITP at the individual level? | NO DATA |  | 0 | 0 | NA |
| **3.2** | How strong is the association between Zika infection and GBS/ITP at the population level? | Ecological study/outbreak report (2): [18, 19] Cross-sectional study (1): [20] Case series (1): [8] | Colombia, Brazil, Dominican Republic, El Salvador, Honduras, Suriname, Venezuela | 4 | 0 | [18] compares the reported pre-ZIKV GBS incidence with the incidence during the ZIKV transmission period in seven different countries (Brazil, Colombia, Dominican Republic, El Salvador, Honduras, Suriname and Venezuela). Rate-ratios are significantly higher for all countries, ranging from 2.0 (95% CI: 1.6-2.6) to 9.8 (95% CI: 7.6-12.5) increase in incidence. [20] describes an increase from an average of 0.67 GBS cases per month to 5.4 cases per month in Rio de Janeiro, Brazil between December 2015 and March 2016. [8] describes surveillance data from Colombia where an increase of GBS cases was noted during the ZIKV outbreak from 20 cases per month to 90 cases per month. [19] describes an increase in cases in Colombia as well. data is provided in: <http://www.ins.gov.co/boletin-epidemiologico/Boletn%20Epidemiolgico/2016%20Bolet%C3%ADn%20epidemiol%C3%B3gico%20semana%209.pdf> |
| **Exclusion of alternatives** | | | | | | |
| **4.1** | Have other explanations/confounders of the association between Zika and GBS/ITP been excluded, such as other infections? | Case series (5): [1, 4, 5, 7, 22] [8] Case report (2): [14, 15] Ecological study/outbreak report (1): [18] | Ecuador, Martinique, Suriname, Guadeloupe, El Salvador, Colombia, Dominican Republic, El Salvador, Honduras, Suriname, Venezuela, Brazil | 9 | 3 | Other infections were assessed in 18 studies. In all these studies DENV infection was assessed [1-5, 7-9, 12-17, 21, 22]. Due to cross-reactivity in serological tests, DENV could not always be excluded with certainty. In three studies previous Campylobacter infection was excluded [4, 9, 17] and in twelve studies CHIKV infection was assessed [1-3, 7, 12, 13, 15-17, 21, 22]. [18] shows that previous cycles of DENV circulation did not result in an increase in reported cases of GBS in Bahia, Brazil. |
| **4.2** | Have other explanations/confounders of the association between Zika and GBS/ITP been excluded, such as vaccines? | Case series (1): [5] | Guadeloupe | 1 | 0 | Vaccination status was assessed in one study [13]. |
| **4.3** | Have other explanations/confounders of the association between Zika and GBS/ITP been excluded, such as underlying systemic disease? | Case series (3): [5, 6, 22] Case report (2): [14, 21] | Martinique, Guadeloupe, Colombia, New Zealand, Ecuador | 5 | 0 | Explanations/confounders of the association between ZIKV and GBS/ITP, such as underlying systemic disease, were excluded in 6 publications [5, 6, 12, 14, 21]. |
| **4.4** | Have other explanations/confounders of the association between Zika and GBS/ITP been excluded, such as concomitant medication, drugs or other chemicals? | Case series (1): [6] Case report (1): [21] | Colombia, New Zealand | 2 | 0 | In [6] 4/19 patients (21%) had occupational exposure to organic solvents. In [21] drug use was excluded. |
| **Cessation** | | | | | | |
| **5.1** | Does the intentional prevention/removal/elimination of Zika infection in individuals, e.g. by insect repellents, lead to a reduction in cases with GBS/ITP? | NO DATA |  | 0 | 0 | NA |
| **5.2** | Does the intentional removal/elimination/prevention of Zika at population-level, e.g. by vector control, lead to a reduction in cases with GBS/ITP? | NO DATA |  | 0 | 0 | NA |
| **5.3** | Does a natural removal/elimination/prevention of Zika at population-level, e.g. increase in immune individuals or decrease in vector abundance lead to a reduction in cases with GBS/ITP? | Case series (1):   [8]  Ecological study/outbreak report (1): [18] | Brazil, Colombia, Dominican Republic, El Salvador, Honduras, Suriname, Venezuela | 2 | 0 | [18] shows in seven different countries (Brazil, Colombia, Dominican Republic, El Salvador, Honduras, Suriname and Venezuela) that GBS incidence goes down with ZIKV incidence. [8] confirms these findings for Colombia. |
| **Dose response** | | | | | | |
| **6.1** | Are the risk and the clinical severity of GBS/ITP associated with viral titres or viral load in the urine? | NO DATA |  | 0 | 0 | NA |
| **Animal experiments** | | | | | | |
| **7.1** | Does inoculation of animals with Zika virus lead to an autoimmune reaction resulting in peripheral neuropathy or thrombocytopenia? | NO DATA |  | 0 | 0 | NA - difficult to answer, since almost all mouse models are in immunosuppressed animals. No experiments in other species (non-human primates for example) are reported. |
| **7.2** | Do other animal experiments support the association of Zika infection and GBS/ITP? | NO DATA |  | 1 | 0 | NA |
| **Analogy** | | | | | | |
| **8.1** | Do other flaviviruses or arboviruses cause GBS/ITP and by which mechanism(s)? | NO DATA |  | 0 | 0 | NA |
| **8.2** | Do other pathogens cause GBS/ITP and by which mechanism(s)? | NO DATA |  | 0 | 0 | NA |
| **8.3** | Which pathogen or host factors facilitate the development of GBS/ITP? | NO DATA |  | 0 | 0 | NA |
| **Specificity** | | | | | | |
| **9.1** | Are there pathological findings in cases with GBS/ITP that are specific for Zika infection? | NO DATA |  | 0 | 0 | NA |
| **Consistency** | | | | | | |
| **10.1** | Is the association between Zika cases and cases with GBS/ITP consistently found across different geographical regions? | Ecological study/outbreak report (4): [18, 23-25] Cross-sectional study (1): [20] | French Southern Territories, Brazil, Colombia, Dominican Republic, El Salvador, Honduras, Suriname, Venezuela | 5 | 0 | Surveillance reports from WHO [24] and PAHO [25] confirm the occurrence of GBS likely linked to ZIKV in 14 countries. In these countries, an increase in incidence of GBS cases was reported, with at least one GBS case with confirmed Zika virus infection (Brazil, Colombia, Dominican Republic, El Salvador, French Guiana, French Polynesia, Guadeloupe, Guatemala, Honduras, Jamaica, Martinique, Puerto Rico, Suriname and Venezuela). WHO classified seven countries as countries with no increase in GBS incidence reported, but at least one GBS case with confirmed Zika virus infection (Bolivia, Costa Rica, Grenada, Haiti, Mexico, Panama and Saint Martin). [18] confirms the increase of GBS likely due to ZIKV in seven different countries (Brazil, Colombia, Dominican Republic, El Salvador, Honduras, Suriname and Venezuela). |
| **10.2** | Is the association between Zika cases and cases with GBS/ITP consistently found across different populations/subpopulations? | Ecological study/outbreak report (2): [18, 23]  Cross-sectional study (1): [20] | French Southern Territories, Brazil, Brazil, Colombia, Dominican Republic, El Salvador, Honduras, Suriname, Venezuela | 3 | 0 | [18] Shows that incidence of ZIKV related GBS increases as age increases. [20, 23] report on the occurrence of GBS in different regions (French Southern Territories, Brazil). |
| **10.3** | Is the association between Zika cases and cases with GBS/ITP consistently found across different Zika lineages/strains? | NO DATA |  | 0 | 0 | NA |
| **10.4** | Is the association between Zika cases and cases with GBS/ITP consistently found across different study designs? | NO DATA |  | 0 | 0 | NA |

# References

1. Zambrano H, Waggoner JJ, Almeida C, Rivera L, Benjamin JQ, Pinsky BA. Zika Virus and Chikungunya Virus CoInfections: A Series of Three Cases from a Single Center in Ecuador. Am J Trop Med Hyg. 2016;95(4):894-6. doi: 10.4269/ajtmh.16-0323. PubMed PMID: 27402518; PubMed Central PMCID: PMC5062796.

2. Dirlikov E, Major CG, Mayshack M, Medina N, Matos D, Ryff KR, et al. Guillain-Barre Syndrome During Ongoing Zika Virus Transmission - Puerto Rico, January 1-July 31, 2016. MMWR Morb Mortal Wkly Rep. 2016;65(34):910-4. doi: 10.15585/mmwr.mm6534e1. PubMed PMID: 27584942.

3. de Laval F, Matheus S, Maquart M, Yvrard E, Barthes N, Combes C, et al. Prospective Zika virus disease cohort: systematic screening. Lancet. 2016;388(10047):868. doi: 10.1016/S0140-6736(16)31429-5. PubMed PMID: 27597462.

4. Langerak T, Yang H, Baptista M, Doornekamp L, Kerkman T, Codrington J, et al. Zika Virus Infection and Guillain-Barre Syndrome in Three Patients from Suriname. Front Neurol. 2016;7(11):233. doi: 10.3389/fneur.2016.00233. PubMed PMID: 28066317; PubMed Central PMCID: PMC5177614.

5. Boyer Chammard T, Schepers K, Breurec S, Messiaen T, Destrem AL, Mahevas M, et al. Severe Thrombocytopenia after Zika Virus Infection, Guadeloupe, 2016. Emerg Infect Dis. 2017;23(4):696-8. doi: 10.3201/eid2304.161967. PubMed PMID: 27997330; PubMed Central PMCID: PMC5367410.

6. Arias A, Torres-Tobar L, Hernandez G, Paipilla D, Palacios E, Torres Y, et al. Guillain-Barre syndrome in patients with a recent history of Zika in Cucuta, Colombia: A descriptive case series of 19 patients from December 2015 to March 2016. J Crit Care. 2017;37:19-23. doi: 10.1016/j.jcrc.2016.08.016. PubMed PMID: 27610587.

7. do Rosario MS, de Jesus PA, Vasilakis N, Farias DS, Novaes MA, Rodrigues SG, et al. Guillain-Barre Syndrome After Zika Virus Infection in Brazil. Am J Trop Med Hyg. 2016;95(5):1157-60. doi: 10.4269/ajtmh.16-0306. PubMed PMID: 27645785; PubMed Central PMCID: PMC5094232.

8. Parra B, Lizarazo J, Jimenez-Arango JA, Zea-Vera AF, Gonzalez-Manrique G, Vargas J, et al. Guillain-Barre Syndrome Associated with Zika Virus Infection in Colombia. N Engl J Med. 2016;375(16):1513-23. doi: 10.1056/NEJMoa1605564. PubMed PMID: 27705091.

9. Langerak T, Yang H, Baptista M, Doornekamp L, Kerkman T, Codrington J, et al. Zika Virus Infection and Guillain-Barre Syndrome in Three Patients from Suriname. Front Neurol. 2016;7(DEC):233. doi: 10.3389/fneur.2016.00233. PubMed PMID: 28066317; PubMed Central PMCID: PMC5177614.

10. Septfons A, Leparc-Goffart I, Couturier E, Franke F, Deniau J, Balestier A, et al. Travel-associated and autochthonous Zika virus infection in mainland France, 1 January to 15 July 2016. Euro Surveill. 2016;21(32):0. doi: 10.2807/1560-7917.ES.2016.21.32.30315. PubMed PMID: 27542120; PubMed Central PMCID: PMC4998503.

11. Walker WL, Lindsey NP, Lehman JA, Krow-Lucal ER, Rabe IB, Hills SL, et al. Zika Virus Disease Cases - 50 States and the District of Columbia, January 1-July 31, 2016. MMWR Morb Mortal Wkly Rep. 2016;65(36):983-6. doi: 10.15585/mmwr.mm6536e5. PubMed PMID: 27631604.

12. Medina MTEJD, Lorenzana I, Medina-Montoya M, Alvarado D, De Bastos M, Fontiveros S, et al. Zika virus associated with sensory polyneuropathy. Journal of the Neurological Sciences. 2016;369(30):271-2.

13. Fabrizius RG, Anderson K, Hendel-Paterson B, Kaiser RM, Maalim S, Walker PF. Guillain-Barre Syndrome Associated with Zika Virus Infection in a Traveler Returning from Guyana. Am J Trop Med Hyg. 2016;95(5):1161-5. doi: 10.4269/ajtmh.16-0397. PubMed PMID: 27807296; PubMed Central PMCID: PMC5094233.

14. Zea-Vera AF, Parra B. Zika virus (ZIKV) infection related with immune thrombocytopenic purpura (ITP) exacerbation and antinuclear antibody positivity. Lupus. 2017;26(8):890-2. doi: 10.1177/0961203316671816. PubMed PMID: 27694629.

15. Fontes CA, Dos Santos AA, Marchiori E. Magnetic resonance imaging findings in Guillain-Barre syndrome caused by Zika virus infection. Neuroradiology. 2016;58(8):837-8. doi: 10.1007/s00234-016-1687-9. PubMed PMID: 27067205.

16. Skrove JL, Salhab J, Colella DM, Michel G, Sobrado J, Almeida M. Zika virus: A rare case with presenting symptoms of gastroenteritis and transaminitis with subsequent progression to guillain-barre syndrome. American Journal of Gastroenterology. 2016;111:S975-S6. doi: 10.1038/ajg.2016.374.

17. Anaya JM, Rodriguez Y, Monsalve DM, Vega D, Ojeda E, Gonzalez-Bravo D, et al. A comprehensive analysis and immunobiology of autoimmune neurological syndromes during the Zika virus outbreak in Cucuta, Colombia. J Autoimmun. 2017;77:123-38. doi: 10.1016/j.jaut.2016.12.007. PubMed PMID: 28062188.

18. Dos Santos T, Rodriguez A, Almiron M, Sanhueza A, Ramon P, de Oliveira WK, et al. Zika Virus and the Guillain-Barre Syndrome - Case Series from Seven Countries. N Engl J Med. 2016;375(16):1598-601. doi: 10.1056/NEJMc1609015. PubMed PMID: 27579558.

19. Machado-Alba JE, Machado-Duque ME, Gaviria-Mendoza A, Orozco-Giraldo V. Diagnosis of neurological disorders and the Zika virus epidemic in Colombia 2014 -2016. Int J Infect Dis. 2016;51:133-4. doi: 10.1016/j.ijid.2016.09.010. PubMed PMID: 27637419.

20. Ferreira da Silva IR, Frontera JA, Moreira do Nascimento OJ. News from the battlefront: Zika virus-associated Guillain-Barre syndrome in Brazil. Neurology. 2016;87(15):e180-e1. doi: 10.1212/WNL.0000000000003024. PubMed PMID: 27421544.

21. Siu R, Bukhari W, Todd A, Gunn W, Huang QS, Timmings P. Acute Zika infection with concurrent onset of Guillain-Barre Syndrome. Neurology. 2016;87(15):1623-4. doi: 10.1212/WNL.0000000000003038. PubMed PMID: 27466468.

22. Chraibi S, Najioullah F, Bourdin C, Pegliasco J, Deligny C, Resiere D, et al. Two cases of thrombocytopenic purpura at onset of Zika virus infection. J Clin Virol. 2016;83(34):61-2. doi: 10.1016/j.jcv.2016.08.299. PubMed PMID: 27596376.

23. Daudens-Vaysse E, Ledrans M, Gay N, Ardillon V, Cassadou S, Najioullah F, et al. Zika emergence in the French Territories of America and description of first confirmed cases of Zika virus infection on Martinique, November 2015 to February 2016. Euro Surveill. 2016;21(28):0. doi: 10.2807/1560-7917.ES.2016.21.28.30285. PubMed PMID: 27447300.

24. World Health Organization. WHO | Zika situation report 05-01-2017 2017 [Access Date:18/12/2017]. Available from: <http://www.who.int/emergencies/zika-virus/situation-report/05-january-2017/en/>.

25. Pan American Health Organization. Epidemiological Update. Zika virus infection - 12 January 2017 2017 [Access Date:20/11/2017]. Available from: <http://www.paho.org/hq/index.php?option=com_docman&task=doc_download&gid=37671&lang=en>.
