# Supplementary material for: Zika virus infection as a cause of congenital brain abnormalities and Guillain-Barré syndrome: From systematic review to living systematic review
Source: F1000Res. 2018 Feb 15;7:196. [Version 1] doi: 10.12688/f1000research.13704.1 (PMC6290976; doi:10.12688/f1000research.13704.1)
Supplement: Supplementary file 5 [file f1000research-7-14886-s0004.tgz › 27bf5125-a9ac-4d35-875c-6fa10af2d555.docx]

Supplementary File 2 – LSR automation methodology

# Workflow

We distinguish searching, deduplication of results, screening, data extraction, data synthesis and data output as different steps in the review process (Figure 1, main text). Searching is the retrieval of publications according to a prespecified search strategy and from prespecified information sources such as medical databases of references defined in PROSPERO CRD42016036693 [1]. Deduplication consists of retaining unique references after combining the results from all different sources are combined. Screening is the process where reviewers assess relevance for inclusion of the publication based on title and abstract and on full text. Relevant items are retained. Data extraction is the process where reviewers extract relevant information according to predefined protocol. Screening and extraction is performed by one reviewer and verified by a second reviewer. In case of disagreement, the dispute is settled by the involvement of a third reviewer. The extracted data is used to construct tables and figures, which are incorporated into a written report.

# Automation

## Searching and deduplication

For living systematic reviews, periodic updating of the search results is key. Deduplicating with high specificity and sensitivity is time consuming and thus burdensome to reviewers [2]. Although reference managers such as Endnote partly automate the deduplication process, manual verification is needed to prevent a high number of false duplicates [3, 4].

Here, we retrieve search results automatically using application programming interface (API) connections to the different information sources. Retrieved results are uniformly formatted to allow comparison. Deduplication is performed based on a rule based algorithm that compares different bibliographical fields. Unique new records are imported into a database and the review team is notified by email of the new entries that are ready for screening.

## Data aggregation and data output to a document

A central database is used for screening, extraction and citation management. The database is accessed in R [5] for data analysis. Using an Rmarkdown script [6], we can export the bibliographic information from our database to BibTex format, allowing the integration of citations. Within the same Rmarkdown script the analyses and formatting of the document or publication is specified. This allows us to automatically update the outputted document as new citations are added or data are extracted.

# References

1. Low N, Krauer F, Riesen M. Causality framework for Zika virus and neurological disorders: systematic review protocol. PROSPERO 2016 CRD42016036693 2016 [Access Date:18/12/2017]. Available from: <http://www.crd.york.ac.uk/PROSPERO/display_record.php?ID=CRD42016036693>.

2. Rathbone J, Carter M, Hoffmann T, Glasziou P. Better duplicate detection for systematic reviewers: evaluation of Systematic Review Assistant-Deduplication Module. Syst Rev. 2015;4(1):6. doi: 10.1186/2046-4053-4-6. PubMed PMID: 25588387; PubMed Central PMCID: PMC4320616.

3. Qi X, Yang M, Ren W, Jia J, Wang J, Han G, et al. Find duplicates among the PubMed, EMBASE, and Cochrane Library Databases in systematic review. PLoS One. 2013;8(8):e71838. doi: 10.1371/journal.pone.0071838. PubMed PMID: 23977157; PubMed Central PMCID: PMC3748039.

4. Bramer WM, Giustini D, de Jonge GB, Holland L, Bekhuis T. De-duplication of database search results for systematic reviews in EndNote. J Med Libr Assoc. 2016;104(3):240-3. doi: 10.3163/1536-5050.104.3.014. PubMed PMID: 27366130; PubMed Central PMCID: PMC4915647.

5. R. Core Team. R: A Language and Environment for Statistical Computing. Vienna, Austria: R Foundation for Statistical Computing; 2017 2017.

6. Allaire JJ, Cheng J, Xie Y, McPherson J, Chang W, Allen J, et al. rmarkdown: Dynamic Documents for R2017 2017.
