# Supplementary material for: Zika virus infection as a cause of congenital brain abnormalities and Guillain-Barré syndrome: From systematic review to living systematic review
Source: F1000Res. 2018 Feb 15;7:196. [Version 1] doi: 10.12688/f1000research.13704.1 (PMC6290976; doi:10.12688/f1000research.13704.1)
Supplement: Supplementary file 6 [file f1000research-7-14886-s0005.tgz › 551d0ee1-1c41-40aa-ab84-da2dfcab9498.docx]

# **Supplementary Text 3 – Search strategy update 2**

Amended search strategy for update 2 and later of “Zika Virus Infection as a Cause of Congenital Brain Abnormalities and Guillain–Barré Syndrome: Systematic Review”.

## Embase

| 1 | zika virus/ |
| --- | --- |
| 2 | zikv.ti,ab. or (zika virus).ti,ab. |
| 3 | congenital disorder/ or fetus/ or mother/ or Guillain Barre syndrome/ |
| 4 | (microcephaly or congenital or fetal or foetal or infant or newborn or guillain$).ti,ab. |
| 5 | Epidemiologic Studies/ OR exp Case-Control Studies/ OR exp Cohort Studies/ OR Cross-Sectional Studies/ OR case report/ OR case series/ |
| 6 | (case report).ab,ti. OR (epidemiologic adj (study or studies)).ab,ti. OR case control.ab,ti. OR (cohort adj (study or studies)).ab,ti. OR cross sectional.ab,ti. OR cohort analy$.ab,ti.OR (follow up adj (study or studies)).ab,ti. OR longitudinal.ab,ti. OR retrospective$.ab,ti. OR prospective$.ab,ti. OR (observ$ adj3 (study or studies)).ab,ti. |
| 7 | 1 or 2 |
| 8 | 3 or 4 |
| 9 | 5 or 6 |
| 10 | 7 and 8 and 9 |

## Pubmed

| 1 | "Zika Virus"[Mesh] |
| --- | --- |
| 2 | zikv[tiab] or zika virus[tiab] |
| 3 | Congenital, Hereditary, and Neonatal Diseases and Abnormalities[mesh] or fetus[mesh] or mother[mesh] or Guillain Barre syndrome[mesh] |
| 4 | microcephaly[tiab] or congenital[tiab] or fetal[tiab] or foetal[tiab] or infant[tiab] or newborn[tiab] or guillain*[tiab] |
| 5 | "Case Reports" [Publication Type] OR "Epidemiologic Studies"[Mesh] OR "Case-Control Studies"[Mesh] OR "Cohort Studies"[Mesh] OR "Cross-Sectional Studies"[Mesh] |
| 6 | case report[tiab] OR epidemiologic study[tiab] OR case control[tiab] OR cohort study[tiab] OR cross sectional[tiab] OR cohort analy*[tiab] OR follow up study[tiab] OR longitudinal[tiab] OR retrospective*[tiab] OR prospective*[tiab] OR observ* study[tiab] |
| 7 | 1 or 2 |
| 8 | 3 or 4 |
| 9 | 5 or 6 |
| 10 | 7 and 8 and 9 |

## Lilacs

(tw:(Zikv) OR tw:(zika virus)) AND (tw:(microcephaly) OR tw:(congenital) OR tw:(fetal) OR tw:(foetal) OR tw:(infant) OR tw:(newborn) OR tw:(guillain*)) AND (tw:(case report) OR tw:(epidemiologic study) OR tw:(case control) OR tw:(cohort study) OR tw:(cross sectional) OR tw:(cohort analy*) OR tw:(follow up study) OR tw:(longitudinal) OR tw:(retrospective*) OR tw:(prospective*) OR tw:(observ* study))
