# Supplementary material for: Zika virus infection as a cause of congenital brain abnormalities and Guillain-Barré syndrome: From systematic review to living systematic review
Source: F1000Res. 2018 Feb 15;7:196. [Version 1] doi: 10.12688/f1000research.13704.1 (PMC6290976; doi:10.12688/f1000research.13704.1)
Supplement: Peer review history

# Peer review: Referee response for version 1

**DOI:** 10.5256/f1000research.14886.r31780

**Published:** 2018-12-10

**Authors:** Alfonso Rodriguez-Morales

**Affiliations:** 1 Faculty of Health Sciences, Universidad Tecnológica de Pereira, Pereira, Colombia

Certainly, a systematic review (SR) on a such highly relevant topic, as Zika association with congenital brain abnormalities and Guillain-Barré syndrome. However, I should suggest first, to include other Zika congenital associated abnormalities, as we have the congenital Zika syndrome (CZS) but also extra-Central Nervous System (CNS) abnormalities.

 The idea to have a living SR is excellent, however very compromising. Right now, this first version, requires urgently to be updated. As the date of update was till January 2017, as a first update.

I have read this submission. I believe that I have an appropriate level of expertise to confirm that it is of an acceptable scientific standard.

# Peer review: Referee response for version 1

**DOI:** 10.5256/f1000research.14886.r34944

**Published:** 2018-07-03

**Authors:** Hugh J. Willison

**Affiliations:** 1 Institute of Infection, Immunity and Inflammation, University of Glasgow, Glasgow, UK

This is a systematic review looking at the strength of evidence supporting the link between Zika virus (ZIKV) infection and neurological complications. The intention is to create a living review that is constantly updated as new publications arise, allowing researchers access to a data resource that keeps pace with the rapidly advancing evidence in the field. This is an extremely important initiative if it can provide truly up to date (i.e. as fast as a pubmed or similar search) and appropriately sifted information for those wishing to conduct spot checks on the current status of neuro-ZIKV.

 From an aspirational perspective I support it strongly, but does it work in practice? Setting aside many  complex methodological considerations that this article raises, how useful is such a resource and who is it there to serve? We would all welcome tailor-made and up-to-date information at our fingertips to help navigate the mountain and minefield of information on ZIKV. I suspect that whilst this review will be widely consulted by those interested in neuro-ZIKV, from policy-makers to researchers, we will still refer back to primary publication for detailed up-to-date information in our specialist areas. At least it will provide a generic overview that facilitates more in depth access to and analysis of the primary data.

I have read this submission. I believe that I have an appropriate level of expertise to confirm that it is of an acceptable scientific standard.

# Peer review: Referee response for version 1

**DOI:** 10.5256/f1000research.14886.r33196

**Published:** 2018-05-04

**Authors:** Katrina J. Sullivan

**Affiliations:** 1 Clinical epidemiology program, Ottawa Hospital Research Institute, Ottawa, ON, Canada

This systematic review assessed the causality of Zika virus in the development of congenital brain abnormalities and Guillain-Barre syndrome. To accomplish this, the 10 dimensions of casual associations were reviewed, and evidence obtained for each dimension were narratively reviewed. With the addition of 101 new studies, the authors were able to conclusively establish the causal association between Zika and adverse congenital outcomes. However, not all dimensions could be assessed concerning Guillain-Barre syndrome, even with the additional of new studies within this update. As a result, there is limited understanding within this field of research for Zika virus.

 Thank you for allowing me to review this article. Overall it was well written, and did a good job addressing all aspects of it's complex objective. Please find below my comments:

 For the methods I would have liked to see more information. This review should be able to stand on it's own without the reader having to find the baseline review to find important methodological information:

1. I would have liked to see inclusion/exclusion criteria for the study (something that could easily be in a figure or table to save room).
2. Please include your search strategy as a supplementary file (just Embase would be sufficient). It sounds like a very simple search the way you've described it in the methods. Also would have liked more information about who designed the search (e.g. information specialist, librarian, the researchers) and if it was peer reviewed using PRESS. Finally, in the "analogy" section of the results you indicate this wasn't reviewed in the baseline review as the search strategy didn't include terms for it. This indicates to me that you must have updated your search between the baseline review and the update, but you don't say this in your methods.
3. There is no assessment for methodological quality, which you do address in your limitations. I think this is an important step when you're including different study designs (although can also be challenging for the same reason). Probably not appropriate to give the same weight to a case report as a cross-sectional study, or an animal experiment and an epidemiological study.  

General:

1. I find Table 1 a bit difficult to read, maybe because instinctively I'd expect the column for "U1, N" to be the total number of studies in the update, rather than just the new studies added with this update. This only becomes obvious when you look at rows like "case series" and you see the U1 has less studies than BR. If you keep this structure, it would be nice to add another column that has total N (as it was the total N that you're drawing conclusion from, the BR and U1 don't stand alone).

Living Systematic Review:

  

- Do the authors provide a clear rationale for the living systematic review? *YES, it meets all criteria for doing a living systematic review (priority question for decision making, important level of uncertainty in the evidence, emerging evidence that will likely impact conclusions) *
- Do the authors clearly state how this will be maintained as a living systematic review? *Partly - While they detail the process of U1-U3, the time for search, between updates, etc. changes with each update (as they anticipate the technology getting better). Living systematic reviews require explicit decisions on how often searching, data extraction, analysis, etc. will be performed.*

I feel this review is premature in saying that it is transitioned to a living systematic review. The intention of the living systematic review is to provide the reader with up to date evidence so the lag between the search and publication is minimize. The authors even define a living systematic review as "a systematic review that is continually updated, incorporating relevant new evidence as it becomes available". The search for this study ended in January 2017, and technically is out of date even for a regular systematic review (which we try to publish within a year). The authors say that they plan to update the review twice per year with formal peer review updates, but if that was the case then another 2 reviews should have been done since this date. Additionally, they authors indicate that only module 1 and part of module 3 of their living systematic review automation were in place by December 2017, meaning this wasn't used for this update.

 Figure 2 already looks to be out of date - in it they propose that U2 would be published by Feb 2018. If they were able to accomplish this then U2 might have been more appropriate to call a living systematic review (where search looks to be from Jan to Dec 2017, and publication is Feb 2018).  

 I think it would have been more appropriate to publish this as an update of the systematic review, publish a protocol for how they were going to transition to a living systematic review (where Figures 1 and 2 would be included), and then treat U2 as their 'baseline" living systematic review, at which point the "living" aspect of the review is turned on and updates are given every 6 months (as they proposed).  

**Do the authors provide a clear rationale for the Living Systematic Review?**

 Yes

**Do the authors clearly state how this will be maintained as a Living Systematic Review?**

 Partly

I have read this submission. I believe that I have an appropriate level of expertise to confirm that it is of an acceptable scientific standard, however I have significant reservations, as outlined above.
